# $\mathscr {S}\mathscr {E}\mathscr {I}\mathscr {A}\mathscr {R}\mathscr {S}$ model for analyzing $\mathscr {C}\mathscr {O}\mathscr {V}\mathscr {I}\mathscr {D}$-19 pandemic process via $\uppsi$-Caputo fractional derivative and numerical simulation

**DOI:** 10.1038/s41598-024-51415-x

**Published:** 2024-01-06

**Authors:** Behnam Mohammadaliee, Vahid Roomi, Mohammad Esmael Samei

**Affiliations:** 1 Department of Mathematics, Azarbaijan Shahid Madani University, Tabriz, Iran; 2 Insurance Research Center, Tehran, Iran; 3 Department of Mathematics, Faculty of Basic Science, Bu-Ali Sina University, Hamedan, 65178-38695 Iran

**Keywords:** Biological techniques, Structural biology, Systems biology, Environmental sciences, Diseases, Health care, Health occupations, Medical research, Risk factors, Mathematics and computing

## Abstract

The objective of this study is to develop the $\mathscr {S}\mathscr {E}\mathscr {I}\mathscr {A}\mathscr {R}\mathscr {S}$ epidemic model for $\mathscr {C}\mathscr {O}\mathscr {V}\mathscr {I}\mathscr {D}$-${\textbf {19}}$ utilizing the $\uppsi$-Caputo fractional derivative. The reproduction number ($\breve{\mathscr {R}}_0$) is calculated utilizing the next generation matrix method. The equilibrium points of the model are computed, and both the local and global stability of the disease-free equilibrium point are demonstrated. Sensitivity analysis is discussed to describe the importance of the parameters and to demonstrate the existence of a unique solution for the model by applying a fixed point theorem. Utilizing the fractional Euler procedure, an approximate solution to the model is obtained. To study the transmission dynamics of infection, numerical simulations are conducted by using MatLab. Both numerical methods and simulations can provide valuable insights into the behavior of the system and help in understanding the existence and properties of solutions. By placing the values $\texttt{t}$, $\ln (\texttt{t})$ and $\sqrt{\texttt{t}}$ instead of $\uppsi$, the derivatives of the Caputo and Caputo–Hadamard and Katugampola appear, respectively, to compare the results of each with real data. Besides, these simulations specifically with different fractional orders to examine the transmission dynamics. At the end, we come to the conclusion that the simulation utilizing Caputo derivative with the order of 0.95 shows the prevalence of the disease better. Our results are new which provide a good contribution to the current research on this field of research.

## Introduction

The initial cases of the novel corona virus (nCoV) were identified in China in December 2019, and the virus quickly spread to other countries around the world, resulting in a significant number of casualties^1^. The WHO declared it a pandemic on March 11, 2020. Many scientists are trying to find the best way to stop the infection from spreading because it has caused a lot of damage to the world. The $\mathscr {C}$-19 pandemic is a severe acute respiratory disease which no definitive treatment has been found till now^1–3^. The droplets, airborne, and closed contact transmission leads to virus spreads from person to person^4–7^. With the outbreak of the pandemic, predicting the number of infected cases and the termination of the infection are important.

It should be noted that mathematical modeling plays a critical role in understanding, predicting, and controlling the spread of diseases. Unlike integer-order models, which only consider the current state of the system, fractional-order models take into account past states and interactions. This memory effect allows for a more accurate representation of the disease dynamics, as it considers the cumulative impact of previous events and interventions on the current state of the disease.

**Figure 1:**
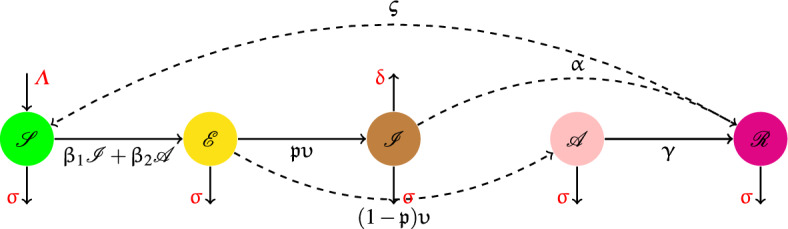
Proposed model $\mathscr {S}\mathscr {E}\mathscr {I}\mathscr {A}\mathscr {R}\mathscr {S}$ for $\mathscr {C}$-19.

In this scientific research, we consider an $\mathscr {S}\mathscr {E}\mathscr {I}\mathscr {A}\mathscr {R}\mathscr {S}$ dynamics model (1) in Fig. 1, such that it individuals are divided into five classes: ($\mathscr {S}$) susceptible individuals; ($\mathscr {E}$) exposed individuals; ($\mathscr {I}$) individuals with symptoms; ($\mathscr {A}$) individuals without symptoms; ($\mathscr {R}$) recovered individuals.

1 $$\begin{aligned} \left\{ \begin{array}{l} \mathscr {S}^\prime = \varLambda - \upbeta _1 \mathscr{S}\mathscr{I} - \upbeta _2 \mathscr{S}\mathscr{A} - \upsigma \, \mathscr {S} + \varsigma \mathscr {R},\\ \mathscr {E}^\prime = \upbeta _1 \mathscr{S}\mathscr{I} - (\upupsilon + \upsigma ) \mathscr {E} + \upbeta _2 \mathscr{S}\mathscr{A},\\ \mathscr {I}^\prime (\texttt{t}) = \mathfrak {p} \upupsilon \mathscr {E} - (\upalpha +\upsigma +\updelta ) \mathscr {I},\\ \mathscr {A}^\prime = ( 1- \mathfrak {p} ) \upupsilon \mathscr {E} - (\upgamma + \upsigma ) \mathscr {A},\\ \mathscr {R}^\prime = \upalpha \mathscr {I} - (\upsigma + \varsigma ) \mathscr {R}+ \upgamma \mathscr {A}. \end{array}\right. \end{aligned}$$

**Figure 2:**
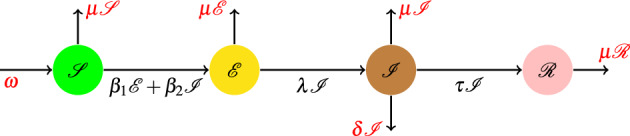
$\mathscr {S}\mathscr {E}\mathscr {I}\mathscr {R}$ model of $\mathscr {C}$-19 in^31^.

New ways of using math of fractional-order have been created to help predict and control the spread of diseases. These models can help us understand how many people will get sick and die, and also help us slow down the spread of the disease^8–13^. Some mathematicians have also focused their efforts on analyzing the different non-linear dynamics of infection-related problems such as epidemics^14–23^. Khan et al. described a mathematical model for dynamics of a novel nCoV (2019) and then developed the it with quarantine and isolation^24,25^. A fractional mathematical model to analyze the pandemic trend of the infection was discussed in^26^. Naik investigated and analyzed a nonlinear fractional-order $\mathscr {S}\mathscr {I}\mathscr {R}$ epidemic model with Crowley–Martin type functional response and Holling type-II treatment rate were established along the memory^22^. Mathematical modeling and analysis of the $\mathscr {C}$-19 epidemic with reinfection and with vaccine availability were examined in^27–30^. The authors in^31^, provided a $\mathscr {S}\mathscr {E}\mathscr {I}\mathscr {R}$ epidemic model, Fig. 2, as form

2 $$\begin{aligned} {\left\{ \begin{array}{ll} \mathscr {S}' = \omega \mu \mathscr {S} - (\beta _1 \mathscr {E} + \beta _2 \mathscr {I}) \texttt{S},\\ \mathscr {E} = (\beta _1\mathscr {E} + \beta _2 \mathscr {I}) \mathscr {S} - (\lambda + \mu ) \mathscr {E},\\ \mathscr {I} = \lambda \mathscr {E} - ( \tau + \mu + \delta ) \mathscr {I},\\ \mathscr {R}= \tau \mathscr {I} - \mu \mathscr {R}, \end{array}\right. } \end{aligned}$$

for the spread of $\mathscr {C}$-19 using the CFD where $\omega = n \times N$, *N* is the total number of individuals and n is the birth rate, $\mu$ is the death rate of people, $\beta _1$, $\beta _2$ are the transmission rate of infection from $\mathscr {E}$ to $\mathscr {S}$, $\mathscr {I}$ to $\mathscr {S}$, respectively, $\lambda$ is the transmission rate of people from $\mathscr {E}$ to $\mathscr {I}$, $\delta$ is the mortality rate due to the disease, and $\tau$ is the rate of recovery of infected people.

**Figure 3:**
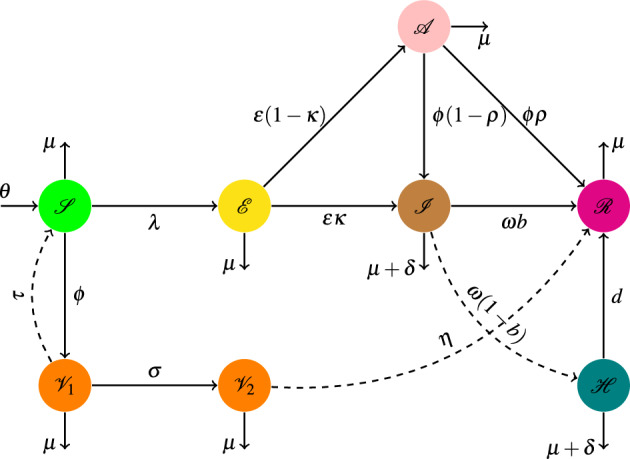
$\mathscr{S}\mathscr{V}_1\mathscr {V}_2\mathscr {E}\mathscr {A}\mathscr {I}\mathscr {H}\mathscr {R}$ model for $\mathscr {C}$-19 in^36^.

Infectious diseases mathematical model is a crucial tool that has been used to study the spreading mechanism of many diseases^32–34^. Gharahasanlou et al. considered the following mathematical biology and dynamical system

3 $$\begin{aligned} \left\{ \begin{array}{l} \mathscr {X}' = \lambda + \alpha \mathscr {X} \left( 1- \frac{\mathscr {X}}{\mathscr {X}_{\max }} \right) d\mathscr {X} -h(\mathscr {X}, \mathscr {Y}, \mathscr {V})\mathscr {V},\\ \mathscr {Y}' = h(\mathscr {X}, \mathscr {Y}, \mathscr {V}) \mathscr {V}- \beta \mathscr {Y} - \gamma \mathscr {Y} \mathscr {Z},\quad \mathscr {V}' = \zeta \mathscr {Y} - \eta \mathscr {V},\quad \mathscr {Z} = \omega \mathscr {Y}\mathscr {Z} - \varpi \mathscr {Z}, \end{array}\right. \end{aligned}$$

under initial conditions $\mathscr {X}(0) > 0$, $\mathscr {Y}(0) > 0$, $\mathscr {Z}(0) > 0$ and $\mathscr {V}(0) > 0$^35^. Recently, in one of the valuable research works, Peter et al.^36^ studied transmission dynamics model, Fig. 3 of $\mathscr {C}$-19 by the following system of the nonlinear differential equation:

4 $$\begin{aligned} \left\{ \begin{array}{l} \mathscr {S}^\prime = \vartheta - \varLambda \mathscr {S} -( \upmu + \varphi ) \mathscr {S}+ \uptau \mathscr {V}_1,\\ \mathscr {V}_1^\prime = \varphi \mathscr {S} - (\uptau + \varsigma + \upmu ) \mathscr {V}_1,\\ \mathscr {V}_2^\prime = \varsigma \mathscr {V}_1 - (\upeta + \upmu ) \mathscr {V}_2,\\ \mathscr {E}^\prime = \varLambda \mathscr {S} - (\upepsilon + \upmu ) \mathscr {E},\\ \mathscr {A}^\prime = \upepsilon ( 1 - \varkappa ) \mathscr {E} - (\upmu + \uppsi ) \mathscr {A},\\ \mathscr {I}^\prime = \upepsilon \varkappa \mathscr {E}+ \uppsi (1 - \varrho ) \mathscr {A} - (\upmu + \updelta + \upomega )\mathscr {I},\\ \mathscr {H}^\prime = \upomega ( 1- b ) \mathscr {I} - (\upmu + \updelta + d) \mathscr {H},\\ \mathscr {R}^\prime = \upomega b \mathscr {I} + \uppsi \varrho \mathscr {A} + d \mathscr {H} + \upeta \mathscr {V}_2- \upmu \mathscr {R}. \end{array}\right. \end{aligned}$$

Musa et al. analyzed a new deterministic co-infection model of $\mathscr {C}$-19^33^. We accept that use these references to influence this phenomena and the consolidation of this marvel and its impacts on the co-dynamics of both illnesses will be of awesome intrigued not as it were to open wellbeing specialists but too to analysts within the field of scientific modeling^9,23,30,33,34^.

The rest of this paper is sorted out within the taking after way. In section “Preliminary definitions”, we review fractional integral and derivative. Then, in section “Model formulation”, the $\mathscr {S}\mathscr {E}\mathscr {I}\mathscr {A}\mathscr {R}\mathscr {S}$ demonstrate of fractional order for the infection transmission is displayed and the balance focuses and their stability are investigated. The existence and asymptotically stability of the equilibrium points are investigated. The sufficient conditions for the persistence of the disease are provided. First, $\breve{\mathscr {R}}_0$ is obtained which determines the stability of equilibria, then model equilibria are determined and their stability analysis is considered by using fractional Routh–Hurwitz stability criterion. The fractional derivative is taken in Caputo sense and the numerical solution of the model is obtained by (8) scheme which involves the memory trace that can capture and integrate all past activity. Meanwhile, by using Lyapunov functional approach, the global dynamics of the endemic equilibrium point is discussed. Further, some numerical simulations are performed to illustrate the effectiveness of the theoretical results obtained. The outcome of the study reveals that the applied (8) scheme is computationally very strong and effective to analyze fractional-order differential equations arising in disease dynamics. The results show that order of the fractional derivative has a significant effect on the dynamic process. The reproduction number is also calculated and its sensitivity is explored. In section “Existence and uniqueness of solution”, we investigate whether there is only one solution for the system. In section “Numerical results”, we use a math method to solve the model and show a math simulation. Eventually, in last section, conclusion is presented.

## Preliminary definitions

Let $\text {J}:= [\grave{\imath }_1, \grave{\imath }_2]$ and consider increasing function $\uppsi :\text {J} \rightarrow \mathbb {R}$ s.t $\uppsi ^\prime (\texttt{t}) \ne 0$, for each $\texttt{t}$. For $\upkappa > 0$, the $\upkappa ^{\text {th}}$ $\uppsi$-Riemann–Liouville fractional ($\uppsi$-RLF) integral for an integrable real function $\upomega$ on $\text {J}$ with respect to $\uppsi$ is illustrated by

5 $$\begin{aligned} \mathscr {I}_{\grave{\imath }_1^{+}}^{\upkappa ;\uppsi } \upomega (\texttt{t}) = \int _{\grave{\imath }_1}^{\texttt{t}} \frac{ \uppsi ^\prime (\xi )}{ ( \tilde{\uppsi }_{\xi }(\texttt{t}) )^{1 - \upkappa }} \frac{\upomega (\xi ) }{ \Gamma (\upkappa )} \, \text {d}\xi , \qquad \tilde{\uppsi }_{\xi }(\texttt{t}): = \uppsi (\texttt{t})-\uppsi (\xi ), \end{aligned}$$

where $\Gamma (\upkappa ) = \int _{0}^{+\infty } e^{-\texttt{t}} \texttt{t}^{\upkappa -1} \, \text {d}\texttt{t}$^37,38^. Let $\uppsi , \upomega \in C^{ \texttt{n}}(\text {J})$ and $\uppsi$ possesses the same properties referred above. The $\upkappa ^{\text {th}}$ $\uppsi$-RLF derivative of $\upomega$ is defined by

$$\begin{aligned} \mathscr {D}_{\grave{\imath }_1^{+}}^{\upkappa ;\uppsi } \upomega ( \texttt{t})&= \mathscr {Z}^{(\texttt{n})} \mathscr {I}_{\grave{\imath }_1^{+}}^{ n-\upkappa ;\uppsi } \upomega ( \texttt{t}) = \mathscr {Z}^{(\texttt{n})} \int _{\grave{\imath }_1}^{ \texttt{t}} \frac{\uppsi ^\prime (\ddot{\xi })}{( \tilde{\uppsi }_{\ddot{\xi }}( \texttt{t}) )^{\upkappa - \texttt{n} +1}} \frac{ \upomega ( \ddot{\xi })}{ \Gamma (\texttt{n}-\upkappa )} \, \text {d}\ddot{\xi }, \qquad \mathscr {Z} = \left( \frac{1}{\uppsi ^\prime (\texttt{t})} \frac{\text {d}}{\text {d}\texttt{t}} \right) , \end{aligned}$$

where $\texttt{n} = [\upkappa ]+1$^37,38^. The $\upkappa ^{\text {th}}$ $\uppsi$-CFD of $\upomega$ is defined by ${}^{\text {C}\!}\mathscr {D}_{\grave{ \imath }_1^{+}}^{\upkappa ;\uppsi } \upomega ( \texttt{t})$ $=\mathscr {I}_{\grave{\imath }_1^{+}}^{ \texttt{n} - \upkappa ; \uppsi } \mathscr {Z}^{(\texttt{n})} \upomega (\texttt{t}),$ where $\texttt{n}=[\upkappa ]+1$ and $\upkappa$ whenever $\upkappa \notin \mathbb {N}$ and $\upkappa \in \mathbb {N}$, respectively^39^. In other words,

6 $$\begin{aligned} {}^{\text {C}\!}\mathscr {D}_{\grave{\imath }_1^{+}}^{\upkappa ; \uppsi } \upomega (\texttt{t}) = \left\{ \begin{array}{cl} \displaystyle \mathscr {Z}^\texttt{n}\upomega (\texttt{t}), &{} \upkappa = \texttt{n} \in \mathbb {N},\\ \displaystyle \int _{\grave{\imath }_1}^{\texttt{t}} \frac{ \uppsi ^\prime (\ddot{\xi })}{ \Gamma (\texttt{n}-\upkappa )} \, \left( \tilde{ \uppsi }_{\ddot{\xi }}(\texttt{t}) \right) ^{\texttt{n}-\upkappa -1} \mathscr {Z}^{ ( \texttt{n})} \upomega (\ddot{\xi })\, \text {d} \ddot{\xi }, &{} \upkappa \notin \mathbb {N}. \end{array}\right. \end{aligned}$$

Extension (6) gives the Caputo and Caputo–Hadamard derivative when $\uppsi (\texttt{t}) = \texttt{t}$ and $\uppsi (\texttt{t})= \ln \texttt{t}$, respectively. The $\uppsi$-CFD of order $\upkappa$ is specified as^39^, Theorem 3,

$$\begin{aligned} {}^{\text {C}\!}\mathscr {D}_{\grave{\imath }_1^{+}}^{ \upkappa ; \uppsi } \upomega (\texttt{t}) = \mathscr {D}_{ \grave{\imath }_1^{+}}^{\upkappa ; \uppsi } \bigg ( \upomega (\texttt{t}) - \sum _{\ell = 0}^{ \mathtt {\texttt{n}}-1} \frac{\mathscr {Z}^{ (\ell )} \upomega ( \grave{\imath }_1 )}{ \ell ! } \left( \tilde{\uppsi }_{ \grave{\imath }_1} (\texttt{t}) \right) ^{\ell } \bigg ). \end{aligned}$$

### Lemma 2.1

(^40^) *Let* $$\upomega \in C^{\texttt{n}} (\text {J})$$. *Then,*

$$\begin{aligned} \mathscr {I}_{\grave{\imath }_1^{+}}^{\upkappa ; \uppsi }~{}^{\text {C}\!}\mathscr {D}_{\grave{\imath }_1^{+}}^{\upkappa ; \uppsi } \upomega (\texttt{t}) = \upomega (\texttt{t}) - \sum _{\ell = 0 }^{\texttt{n}-1} \frac{\mathscr {Z}^{(\ell )} \upomega (\grave{\imath }_1)}{\ell !} \left[ \tilde{\uppsi }_{\grave{\imath }_1} (\texttt{t}) \right] ^{ \ell }, \qquad \forall \, \texttt{t}\in \text {J}, \, \texttt{n}-1<\upkappa <\texttt{n}. \end{aligned}$$

Also, if $\upomega \in C^{\texttt{n} + \texttt{m}}(\text {J} )$ $(\texttt{m}\in \mathbb {N})$, then

7 $$\begin{aligned} \mathscr {Z}^{(\texttt{m})} \left( {}^{\text {C}\!}\mathscr {D}_{\grave{\imath }_1^{+}}^{\upkappa ; \uppsi } \upomega \right) (\texttt{t}) = ~{}^{\text {C}\!}\mathscr {D}_{\grave{\imath }_1^{+}}^{ \upkappa +\texttt{m}; \uppsi }\upomega (\texttt{t}) + \sum _{\ell =0}^{\texttt{m}-1} \frac{ \left[ \tilde{\uppsi }_{ {\grave{\imath }_1} } (\texttt{t}) \right] ^{\ell + \texttt{n}-\upkappa - \texttt{m}}}{\Gamma (\ell + \texttt{n} - \upkappa - \texttt{m}+1) } \mathscr {Z}^{(\ell + \texttt{n})} \upomega ({ \ell _1}). \end{aligned}$$

Watch that in the case of $\mathscr {Z}^{ ( \ell ) } \upomega (\grave{\imath }_1) = 0$, $\texttt{n} \le \ell \le \texttt{n} + \texttt{m}-1$, the relationship

$$\begin{aligned} \mathscr {Z}^{(\texttt{m})}\left( {}^{\text {C}\!}\mathscr {D}_{\grave{\imath }_1^{+}}^{\upkappa ;\uppsi } \upomega \right) (\texttt{t}) = {}^{\text {C}\!} \mathscr {D}_{\grave{\imath }_1^{+}}^{\upkappa +\texttt{m}; \uppsi }\upomega (\texttt{t}), \qquad \texttt{t} \in \text {J}, \end{aligned}$$

can be obtained

### Lemma 2.2

(^40^) *Let* $\upkappa , \upnu >0$ *and* $\upomega \in C(\text {J})$. *Then, for all* $\texttt{t} \in \text {J}$, (i)

$$\begin{aligned} \mathscr {I}_{\grave{\imath }_1^{+}}^{\upkappa ;\uppsi } \big (\mathscr {I}_{ \grave{\imath }_1^{+}}^{\upnu ; \uppsi } \upomega \big )(\texttt{t}) = \mathscr {I}_{ \grave{\imath }_1^{+}}^{\upkappa + \upnu ;\uppsi } \upomega (\texttt{t}), \qquad {}^{\text {C}\!} \mathscr {D}_{\grave{ \imath }_1^{+}}^{\upkappa ;\uppsi } \big ( \mathscr {I}_{\grave{\imath }_1^{+}}^{\upkappa ; \uppsi }\upomega \big ) (\texttt{t}) = \upomega (\texttt{t}); \end{aligned}$$

(ii) *with* $$\Lambda = \upnu + \upkappa -1$$,

$$\begin{aligned} \mathscr {I}_{\grave{\imath }_1^{+}}^{\upkappa ; \uppsi } ( \tilde{\uppsi }_{\grave{\imath }_1}( \texttt{t}))^{\upnu -1} = \frac{\Gamma (\upnu )}{ \Gamma (\upnu +\upkappa )}( \tilde{ \uppsi }_{\grave{\imath }_1} (\texttt{t}))^{\Lambda };\qquad {}^{\text {C}\!}\mathscr {D}_{ \grave{\imath }_1^{+}}^{\upkappa ;\uppsi }( \tilde{ \uppsi }_{ \grave{\imath }_1}(\texttt{ t}))^{\upnu -1} =\frac{\Gamma (\upnu )}{\Gamma (\upnu -\upkappa )}(\tilde{ \uppsi }_{ \grave{\imath }_1} ( \texttt{t}))^{\Lambda }; \end{aligned}$$

(iii) $${}^{\text {C}\!}\mathscr {D}_{\grave{\imath }_1^{+}}^{\upkappa ; \uppsi }( F_{\grave{\imath }_1})^{\ell } = 0, \, \texttt{n} - 1\le \upkappa \le \texttt{n}$$, $$\ell =0,1, \dots ,\texttt{n}-1, \texttt{n}\in \mathbb {N}$$.

### Theorem 2.3

(Banach Contraction Principle^41^) *Let* $\mathbb {W}\ne \varnothing$ *and* $(\mathbb {W},{\rho })$ *be a complete metric space via a contraction* $\varUpsilon : \mathbb {W}\rightarrow \mathbb {W}$ *i.e.*, ${\rho }(\varUpsilon \upomega , \varUpsilon \upomega ^*) \le \upsigma \rho (\upomega , \upomega ^*)$, *for each* $\upomega , \upomega ^* \in \mathbb {W},\, \upsigma \in (0, 1)$. *Then*, $\varUpsilon$ *admits a FP uniquely*.

### Theorem 2.4

(Leray–Schauder^41^) *Let* $\texttt{B}$ *be a bounded convex closed set and* $\texttt{O}$ *be open set contained in* $\texttt{B}$ *of Banach space* $\mathbb {W}$ *with* $0\in \texttt{O}$. *Then, for the continuous and compact mapping* $\varUpsilon : \overline{\texttt{O}} \rightarrow \texttt{B}$, *either* (i) $\varUpsilon$ *admits a FP belonging to* $\bar{\texttt{O}}$; (ii) $\exists \, \upomega \in \partial \texttt{O}$, $0< \upsigma < 1$ *s.t* $\upomega = \upsigma \varUpsilon (\upomega )$.

## Model formulation

Mathematical models play a crucial role in predicting the behavior of viruses and their transmission among individuals during a viral pandemic. These models are essential for understanding how diseases spread in different parts of the world and for effectively managing the outbreak. Various mathematical models, such as the $\mathscr {S}\mathscr {I}\mathscr {R}$, $\mathscr {S}\mathscr {E}\mathscr {I}\mathscr {R}$, $\mathscr {S}\mathscr {C}\mathscr {I}\mathscr {A}\mathscr {R}$, $\mathscr {S}\mathscr {E}\mathscr {I}\mathscr {Q}\mathscr {T}\mathscr {S}$, and $\mathscr {S}\mathscr {E}\mathscr {I}\mathscr {A}\mathscr {R}\mathscr {S}$, etc. are used to evaluate the prevalence of diseases. Based on the data provided by the WHO regarding $\mathscr {C}$-19, there are two categories of individuals infected with the virus: asymptomatic and symptomatic. Both types of individuals can transmit the disease to healthy individuals, and the infected individuals may either recover or succumb to the illness.

We aimed to change the time derivative with the $\uppsi$-CFD in system (1) for the disease in Fig. 1 under the parameters are explained in Table 1, by introducing a parameter $\uptheta$ in the following way

$$\begin{aligned} \left[ \uptheta ^{\upkappa -1}{{}^{\text {C}\!} \mathscr {D}}^{\upkappa ;  {\uppsi }} \mathscr {S}(\texttt{t}) \right] = \left[ \frac{\text {d}}{\text {d}\texttt{t}} \right] = \mathfrak {s}^{-1}. \end{aligned}$$

**Table 1:** The parameters description used in model.

Parameter explanation	Parameter
Birth rate of population	$\varLambda$
Natural death rate	$\upsigma$
Changing rate from $\mathscr {R}$ to $\mathscr {S}$, $\mathscr {E}$ to $\mathscr {I}$ and $\mathscr {A}$	$\varsigma$, $\upupsilon$
Transmission rate of infection from $\mathscr {I}$ to $\mathscr {S}$, $\mathscr {A}$ to $\mathscr {S}$	$\upbeta _1$, $\upbeta _2$
Recovery rate of infected and asymptomatic population	$\upalpha$, $\upgamma$
Mortality rate because to the disease	$\updelta$
Population progress to $\mathscr {I}$	$\mathfrak {p}$

Consequently, the $\mathscr {C}$-19 mathematical model based on fractional derivatives of order $0< \upkappa < 1$ is presented as

8 $$\begin{aligned} \left\{ \begin{array}{l} \uptheta ^{\upkappa -1}{{}^{\text {C}\!}\mathscr {D}}_{\grave{\imath }_1^{+}}^{\upkappa ;  {\uppsi }}\mathscr {S}= \varLambda - \upsigma \, \mathscr {S} + \varsigma \mathscr {R} - \upbeta _1\mathscr {S}(\texttt{t})\mathscr {I}(\texttt{t}) - \upbeta _2 \mathscr {S} \mathscr {A},\\ \uptheta ^{\upkappa -1}{{}^{\text {C}\!}\mathscr {D}}_{\grave{\imath }_1^{+}}^{ \upkappa ; {\uppsi }} \mathscr {E} = \upbeta _1 \mathscr {S} \mathscr {I} - (\upupsilon + \upsigma ) \mathscr {E} + \upbeta _2\mathscr {S} \mathscr {A},\\ \uptheta ^{\upkappa -1}{{}^{\text {C}\!}\mathscr {D}}_{\grave{\imath }_1^{+}}^{\upkappa ; {\uppsi }} \mathscr {I} = \mathfrak {p}\upupsilon \mathscr {E} -(\upalpha +\upsigma +\updelta )\mathscr {I},\\ \uptheta ^{\upkappa -1}{{}^{\text {C}\!}\mathscr {D}}_{\grave{\imath }_1^{+}}^{\upkappa ; {\uppsi }} \mathscr {A} = ( 1-\mathfrak {p})\upupsilon \mathscr {E} - (\upgamma +\upsigma )\mathscr {A},\\ \uptheta ^{\upkappa -1}{{}^{\text {C}\!}\mathscr {D}}_{\grave{\imath }_1^{+}}^{\upkappa ;  { \uppsi }}\mathscr {R} = \upalpha \mathscr {I} -(\upsigma + \varsigma ) \mathscr {R} + \upgamma \mathscr {A}, \end{array}\right. \end{aligned}$$

for $\texttt{t} > 0$ under ICs

$$\begin{aligned} \mathscr {S}(0)=\mathscr {S}_0>0,\quad \mathscr {E}(0) = \mathscr {E}_0>0,\quad \mathscr {I}(0)=\mathscr {I}_0>0,\quad \mathscr {A}(0)=\mathscr {A}_0> 0,\quad \mathscr {R}(0)=\mathscr {R}_0>0. \end{aligned}$$

### Lemma 3.1

*Let closed set is expressed by*

$$\begin{aligned} \tilde{ \uppsi } = \left\{ (\mathscr {S},\mathscr {E},\mathscr {I},\mathscr {A},\mathscr {R})\in \mathbb {R}^+_5 \,:\, \aleph (\texttt{t}) = \mathscr {S}+ \mathscr {E}+ \mathscr {I}+ \mathscr {A}+ \mathscr {R} \le \frac{\varLambda }{\upsigma } \right\} . \end{aligned}$$

*Then* $\tilde{ \uppsi }$ *is positively invariant with respect to system* (8).

### Proof

Add all of the relations in system (8) to get

$$\begin{aligned} \uptheta ^{\upkappa -1}{\mathscr {D}}_{\grave{\imath }_1^{+}}^{\upkappa ;  {\uppsi }} \aleph (\texttt{t})&= \uptheta ^{\upkappa -1} \left( {\mathscr {D}}_{\grave{\imath }_1^{+}}^{\upkappa ; {\uppsi }}\mathscr {S} + {\mathscr {D}}_{\grave{\imath }_1^{+}}^{\upkappa ; {\uppsi }}\mathscr {E} + {\mathscr {D}}_{\grave{\imath }_1^{+}}^{\upkappa ;  {\uppsi }}\mathscr {I} + {\mathscr {D}}_{\grave{\imath }_1^{+}}^{\upkappa ; {\uppsi }} \mathscr {A} + {\mathscr {D}}_{\grave{\imath }_1^{+}}^{\upkappa ; {\uppsi }}\mathscr {R}\right) = \varLambda -\upsigma \aleph (\texttt{t})-\updelta \mathscr {I}. \end{aligned}$$

Thus,

$$\begin{aligned} \uptheta ^{\upkappa -1}{\mathscr {D}}_{\grave{\imath }_1^{+}}^{\upkappa ; {\uppsi }} \aleph (\texttt{t}) \le \varLambda - \upsigma \aleph (\texttt{t})\Rightarrow {\mathscr {D}}_{\grave{\imath }_1^{+}}^{\upkappa ; {\uppsi }} \aleph (\texttt{t}) \le \uptheta ^{1-\upkappa } \varLambda - \uptheta ^{1-\upkappa }\upsigma \aleph (\texttt{t}). \end{aligned}$$

By applying^42^, Theorem 7.2, we conclude

$$\begin{aligned} \aleph (\texttt{t}) \le \aleph (0)\mathscr {E}_\upkappa (-\upsigma \uptheta ^{1 - \upkappa }\texttt{t}^{\upkappa }) + \int _{0}^{\texttt{t}} \varLambda \uptheta ^{1-\upkappa }\rho ^{\upkappa -1} \mathscr {E}_{\upkappa ,\upkappa }(-\upsigma \uptheta ^{1-\upkappa } \rho ^{\upkappa })\text {d}\rho . \end{aligned}$$

Hence,

$$\begin{aligned} \aleph (\texttt{t})&\le \aleph (0) \mathscr {E}_\upkappa (-\upsigma \uptheta ^{1-\upkappa }\texttt{t}^{\upkappa }) + \int _{0}^{\texttt{t}} \varLambda \uptheta ^{1-\upkappa } \rho ^{\upkappa -1} \sum _{\mathfrak {i}=0}^{\infty } \frac{(-1)^{\mathfrak {i}} \upsigma ^{\mathfrak {i}} \uptheta ^{(1-\upkappa ) \mathfrak {i}} \rho ^{\mathfrak {i}\upkappa }}{ \Gamma (\mathfrak {i} \upkappa + \upkappa )}\text {d}\rho \\&= \aleph (0)\mathscr {E}_\upkappa (-\upsigma \uptheta ^{1-\upkappa } \texttt{t}^{\upkappa }) + \varLambda \uptheta ^{1-\upkappa } \sum _{\mathfrak {i}=0}^{\infty } \frac{(-1)^{\mathfrak {i}} \upsigma ^{\mathfrak {i}} \uptheta ^{(1-\upkappa ) \mathfrak {i}}\texttt{t}^{\mathfrak {i}\upkappa + \upkappa }}{\Gamma (\mathfrak {i}\upkappa +\upkappa +1)}\\&= \aleph (0) \mathscr {E}_\upkappa (-\upsigma \uptheta ^{1-\upkappa }\texttt{t}^{\upkappa }) - \frac{\varLambda }{\upsigma }\sum _{\mathfrak {i}=0}^{ \infty } \frac{(-1)^{\mathfrak {i}} \upsigma ^{\mathfrak {i}} \uptheta ^{(1-\upkappa ) \mathfrak {i}} \texttt{t}^{\mathfrak {i} \upkappa }}{\Gamma (\mathfrak {i} \upkappa +1)}\\&=\aleph (0) \mathscr {E}_\upkappa (-\upsigma \uptheta ^{1- \upkappa }\texttt{t}^{\upkappa }) - \frac{\varLambda }{\upsigma } \left( \mathscr {E}_{ \upkappa } \left( -\upsigma \uptheta ^{ 1 -\upkappa }\texttt{t}^{\upkappa -1} \right) \right) = \frac{\varLambda }{\upsigma }+\mathscr {E}_\upkappa (-\upsigma \uptheta ^{1-\upkappa }\texttt{t}^{\upkappa })\bigg (\aleph (0)-\frac{\varLambda }{\upsigma }\bigg ). \end{aligned}$$

Thus, if $\aleph (0) \le \frac{\varLambda }{\upsigma }$, then $\aleph (\texttt{t}) \le \frac{\varLambda }{\upsigma }$ for each positive real number $\texttt{t}$. This completes the proof. $\square$

### Equilibrium points and stability

Equilibrium points (EPs) of system (8) can be determined by solving the following equations.

9 $$\begin{aligned} \left\{ \begin{array}{l} \varLambda -\upbeta _1\mathscr {S} \mathscr {I} - \upbeta _2 \mathscr {S} \mathscr {A} - \upsigma \, \mathscr {S} + \varsigma \mathscr {R}=0,\\ \upbeta _1\mathscr {S} \mathscr {I} + \upbeta _2\mathscr {S} \mathscr {A} - (\upupsilon + \upsigma ) \mathscr {E} =0,\\ \mathfrak {p} \upupsilon \mathscr {E} - (\upalpha +\upsigma +\updelta )\mathscr {I} =0,\\ (1-\mathfrak {p})\upupsilon \mathscr {E} - (\upgamma +\upsigma ) \mathscr {A}=0,\\ \upalpha \mathscr {I}+ \upgamma \mathscr {A} -(\upsigma + \varsigma ) \mathscr {R}=0. \end{array}\right. \end{aligned}$$

Clearly, whenever there is no spread of the disease; i.e., $\mathscr {I}=0$, then a disease-free equilibrium (DFE) is occurred. Hence, the DFE point is obtained as $\mathscr {E}_0 = (\frac{\varLambda }{\upsigma },0,0,0, 0 )$. If $\breve{\mathscr {R}}_0>1$, one can find others EPs of the model by solving (9). Therefore, we obtain the endemic EPs of the model whenever $\mathscr {S}$, $\mathscr {E}$, $\mathscr {I}$, $\mathscr {A}$, $\mathscr {R}$ is against zero, and it is in the form: $\mathscr {E}_1 = \left( \mathscr {S}^{\star },\mathscr {E}^{\star },\mathscr {I}^{\star },\mathscr {A}^{\star },\mathscr {R}^{\star } \right)$, where

$$\begin{aligned} \mathscr {S}^{\star }&=\frac{(\upupsilon + \upsigma ) ( \upalpha + \upsigma + \updelta )( \upgamma + \upsigma )}{\upbeta _1 \mathfrak {p} \upupsilon ( \upgamma +\upsigma ) +\upbeta _2(1-\mathfrak {p})\upupsilon (\upalpha +\upsigma +\updelta )},\\ \mathscr {E}^{\star }&=\frac{(\varLambda -\upsigma \mathscr {S}^{\star })(\upalpha +\upsigma +\updelta )(\upgamma +\upsigma ) (\upsigma +\varsigma )}{(\upupsilon +\upsigma )(\upalpha +\upsigma +\updelta )(\upgamma +\upsigma )(\upsigma +\varsigma ) -\mathfrak {p}\upupsilon \upalpha \varsigma (\upgamma +\upsigma )-(1-\mathfrak {p})\upupsilon \upgamma \varsigma (\upalpha +\upsigma +\updelta )},\\ \mathscr {I}^{\star }&=\frac{\mathfrak {p} \upupsilon \mathscr {E}^{\star }}{(\upalpha +\upsigma +\updelta )}, \quad \mathscr {A}^{\star } =\frac{(1-\mathfrak {p}) \upupsilon \mathscr {E}^{\star }}{(\upgamma +\upsigma )}, \quad \mathscr {R}^{\star } =\frac{\upalpha \mathscr {I}^{\star }+\upgamma \mathscr {A}^{\star }}{\upsigma +\varsigma }. \end{aligned}$$

In order to find $\breve{\mathscr {R}}_0$, the system is considered as ${{}^{\text {C}\!}\mathscr {D}}^{\upkappa ; {\uppsi }}\varPhi (\texttt{t}) =\mathfrak {F}(\varPhi (\texttt{t}))-\mathfrak {V}(\varPhi (\texttt{t}))$, where

$$\begin{aligned} \mathfrak {F}(\varPhi (\texttt{t})) = \uptheta ^{1-\upkappa } \begin{pmatrix} (\upbeta _1\mathscr {I}+\upbeta _2\mathscr {A})\mathscr {S}\\ 0\\ 0\\ \end{pmatrix},\qquad \mathfrak {V}(\varPhi (\texttt{t})) = \uptheta ^{1-\upkappa }\begin{pmatrix} (\upupsilon +\upsigma )\mathscr {E}\\ (\upalpha +\upsigma +\updelta )\mathscr {I} -\mathfrak {p}\upupsilon \mathscr {E} \\ (\upgamma +\upsigma )\mathscr {A} -(1-\mathfrak {p})\upupsilon \mathscr {E} \\ \end{pmatrix}. \end{aligned}$$

At $\mathscr {E}_0$, the Jacobian matrix for $\mathfrak {F}$ and $\mathfrak {V}$ is gotten as

$$\begin{aligned} \mathscr {J}_{\mathfrak {F}} ( \mathscr {E}_0) =\uptheta ^{1-\upkappa } \begin{pmatrix} 0&{} \upbeta _1\frac{\varLambda }{\upsigma }&{} \upbeta _2\frac{\varLambda }{\upsigma }\\ 0&{} 0&{} 0\\ 0&{} 0&{} 0\\ \end{pmatrix},\qquad \mathscr {J}_{\mathfrak {V}}(\mathscr {E}_0) = \uptheta ^{1-\upkappa } \begin{pmatrix} \upupsilon +\upsigma &{} 0&{} 0\\ -\mathfrak {p}\upupsilon &{} \upalpha +\upsigma +\updelta &{} 0\\ -(1-\mathfrak {p})\upupsilon &{} 0&{} \upgamma +\upsigma \\ \end{pmatrix}. \end{aligned}$$

The next-generation matrix for system (8) is as

$$\begin{aligned} \mathscr{F}\mathscr{V}^{-1}=\begin{pmatrix} \frac{\upbeta _1\frac{\varLambda }{\upsigma }\mathfrak {p}\upupsilon }{(\upupsilon +\upsigma ) (\upalpha +\upsigma +\updelta )} +\frac{\upbeta _2\frac{\varLambda }{\upsigma } (1-\mathfrak {p})\upupsilon }{(\upupsilon +\upsigma )(\upgamma +\upsigma )}&{} \frac{\upbeta _1\frac{\varLambda }{\upsigma }}{\upalpha +\upsigma +\updelta }&{} \frac{\upbeta _2\frac{\varLambda }{\upsigma }}{\upgamma +\upsigma }\\ 0&{} 0&{} 0\\ 0&{} 0&{} 0\\ \end{pmatrix}, \end{aligned}$$

and the reproduction number is gotten from $\breve{\mathscr {R}}_0=\uprho (\mathscr{F}\mathscr{V}^{-1})$. Therefore,

$$\begin{aligned} \breve{\mathscr {R}}_0=\frac{\mathfrak {p}\upupsilon \upbeta _1\varLambda }{\upsigma (\upupsilon +\upsigma ) (\upalpha +\upsigma +\updelta )}+\frac{(1-\mathfrak {p})\upupsilon \upbeta _2\varLambda }{\upsigma (\upupsilon +\upsigma )(\upgamma +\upsigma )}. \end{aligned}$$

#### Theorem 3.2

*The DFE point* $\mathscr {E}_0$ *of* (8) *is locally asymptotically stable if* $\breve{\mathscr {R}}_0 < 1$ *and it is unstable if* $\breve{\mathscr {R}}_0 >1$.

#### Proof

The Jacobian matrix of (8) at $\mathscr {E}_0=\left( \mathscr {S}^{0},0,0,0,0 \right)$ is

$$\begin{aligned} \mathscr {J}(\mathscr {E}_0)=\uptheta ^{1-\upkappa }\begin{pmatrix} -\upsigma &{} 0&{} -\upbeta _1\mathscr {S}^{0}&{} -\upbeta _2\mathscr {S}^{0} &{} \varsigma \\ 0&{} -(\upupsilon +\upsigma )&{} \upbeta _1\mathscr {S}^{0}&{} \upbeta _2\mathscr {S}^{0} &{} 0 \\ 0&{} \mathfrak {p}\upupsilon &{} -(\upalpha +\upsigma +\updelta ) &{} 0 &{} 0 \\ 0&{} (1-\mathfrak {p})\upupsilon &{} 0 &{} -(\upgamma +\upsigma ) &{} 0 \\ 0&{} 0&{} \upalpha &{} \upgamma &{} -(\upsigma +\varsigma ) \\ \end{pmatrix}, \end{aligned}$$

where $\mathscr {S}^{0}=~\frac{\varLambda }{\upsigma }$. The characteristic equation $|\mathscr {J}(\mathscr {E}_0)-\uplambda \mathscr {I}|=0$, has two eigenvalues $\uplambda _1 = - \upsigma$ and $\uplambda _2 = - ( \upsigma + \varsigma )$. The remaining three eigenvalues are those of the $3 \times 3$ matrix

$$\begin{aligned} \mathscr {J}_1=\uptheta ^{1-\upkappa } \begin{pmatrix} -(\upupsilon +\upsigma )&{} \upbeta _1\mathscr {S}^{0}&{} \upbeta _2\mathscr {S}^{0}\\ \mathfrak {p}\upupsilon &{} -(\upalpha +\upsigma +\updelta ) &{} 0 \\ (1-\mathfrak {p})\upupsilon &{} 0 &{} -(\upgamma +\upsigma ) \\ \end{pmatrix}. \end{aligned}$$

The characteristic equation takes the form

$$\begin{aligned} \begin{vmatrix} -(\upupsilon +\upsigma +\uplambda )&\upbeta _1\mathscr {S}^{0}&\upbeta _2\mathscr {S}^{0}\\ \mathfrak {p}\upupsilon&-(\upalpha +\upsigma +\updelta +\uplambda )&0 \\ (1-\mathfrak {p})\upupsilon&0&-(\upgamma +\upsigma +\uplambda ) \\ \end{vmatrix}=0. \end{aligned}$$

Thus,

$$\begin{aligned} (\upupsilon +\upsigma +\uplambda ) (\upalpha +\upsigma +\updelta +\uplambda )(\upgamma +\upsigma +\uplambda ) -\upbeta _2 \mathscr {S}^{0}(1-\mathfrak {p})\upupsilon (\upalpha +\upsigma +\updelta +\uplambda ) -\upbeta _1\mathscr {S}^{0}\mathfrak {p}\upupsilon (\upgamma +\upsigma +\uplambda )=0. \end{aligned}$$

Therefore, $$\mathscr {P}(\uplambda ) : =\uplambda ^3+\mathfrak {b}_1\uplambda ^2+\mathfrak {b}_2\uplambda +\mathfrak {b}_3=0$$, where $$\mathfrak {b}_1=\upupsilon +\upsigma + \upalpha + \upsigma + \upgamma + \updelta + \upsigma $$,

$$\begin{aligned} \mathfrak {b}_2&=(\upalpha +\upsigma +\updelta )(\upupsilon +\upsigma )+(\upalpha +\upsigma +\updelta ) (\upgamma +\upsigma )+(\upgamma +\upsigma )(\upupsilon +\upsigma ) -\upbeta _2\mathscr {S}^{0}(1-\mathfrak {p})\upupsilon -\upbeta _1\mathscr {S}^{0}\mathfrak {p}\upupsilon ,\\ \mathfrak {b}_3&=(\upalpha +\upsigma +\updelta )(\upupsilon +\upsigma )(\upgamma +\upsigma ) -(\upalpha +\upsigma +\updelta )\upbeta _2\mathscr {S}^{0}(1-\mathfrak {p})\upupsilon -(\upgamma +\upsigma )\upbeta _1\mathscr {S}^{0}\mathfrak {p}\upupsilon . \end{aligned}$$

It is clear that if $\mathfrak {b}_3$ s greater than zero, it means that $\breve{\mathscr {R}}_0$ is less than one. In addition, if the value of $\breve{\mathscr {R}}_0$ is greater than 1, then $\mathfrak {b}_3$ is less than zero.

From $\lim _{\uplambda \rightarrow \infty } \mathscr {P}(\uplambda )=\infty$, one can conclude that $\mathscr {P}(\uplambda ) = 0$ has a real positive solution, and the EP $\mathscr {E}_0$ is thus unstable. The EP $\mathscr {E}_0$ is locally asymptotically stable whenever $\breve{\mathscr {R}}_0 < 1$. Specifically, we need to prove that in this situation, the equation $\mathscr {P}(\uplambda ) = 0$ only has solutions that are negative or have a negative real part by employing the Routh–Hurwitz criteria. It follows from $\breve{\mathscr {R}}_0 < 1$ that $\mathfrak {b}_3 > 0$. Expressly, $\mathfrak {b}_1 > 0$. The condition $\breve{\mathscr {R}}_0 < 1$ all so gives

$$\begin{aligned} (\upupsilon +\upsigma )(\upgamma +\upsigma )>\upbeta _2 \mathscr {S}^{0}(1-\mathfrak {p})\upupsilon , \qquad (\upalpha +\upsigma +\updelta )(\upupsilon +\upsigma )>\upbeta _1 \mathscr {S}^{0} \mathfrak {p}\upupsilon . \end{aligned}$$

Hence, $\mathfrak {b}_2>0$. Since $(\upupsilon +\upsigma )(\upgamma +\upsigma )>\upbeta _2 \mathscr {S}^{0} (1-\mathfrak {p})\upupsilon$ and $(\upalpha +\upsigma +\updelta )(\upupsilon +\upsigma )> \upbeta _1 \mathscr {S}^{0} \mathfrak {p}\upupsilon$, we get

$$\begin{aligned} \mathfrak {b}_1\mathfrak {b}_2 > (\upupsilon +\upsigma )(\upgamma +\upsigma )(\upalpha +\upsigma +\updelta ). \end{aligned}$$

On the other hand, $\mathfrak {b}_3<(\upupsilon +\upsigma )(\upgamma +\upsigma )(\upalpha +\upsigma +\updelta )$ and therefore, $\mathfrak {b}_1\mathfrak {b}_2>(\upupsilon +\upsigma )(\upgamma +\upsigma )(\upalpha +\upsigma +\updelta )>\mathfrak {b}_3.$ The Routh–Hurwitz yardstick then connotes that the EP $\mathscr {E}_0$ is locally asymptotically stable whenever $\breve{\mathscr {R}}_0$ is less than 1. $\square$

#### Theorem 3.3

*The DFE point* $\mathscr {E}_0$ of (8) *is globally asymptotically stable if* $\breve{\mathscr {R}}_0 \le 1$.

#### Proof

To display the result, define a Lyapunov function as $\mathfrak {L}(\texttt{t})=\vartheta _1\mathscr {E}(\texttt{t})+\vartheta _2\mathscr {I}(\texttt{t}) +\vartheta _3\mathscr {A}(\texttt{t})$, where $\vartheta _1$,$\vartheta _2$ and $\vartheta _3$ are positive constants. The $\uppsi$-CFD of the Lyapunov function is given by,

$$\begin{aligned} {{}^{\text {C}\!}\mathscr {D}}_{\grave{\imath }_1^{+}}^{\upkappa ; {\uppsi }}\mathfrak {L}(\texttt{t})=\vartheta _1{{}^{\text {C}\!}\mathscr {D}}_{\grave{\imath }_1^{+}}^{\upkappa ; {\uppsi }}\mathscr {E}(\texttt{t})+\vartheta _2{{}^{\text {C}\!}\mathscr {D}}_{\grave{\imath }_1^{+}}^{\upkappa ; {\uppsi }}\mathscr {I}(\texttt{t})+\vartheta _3{{}^{\text {C}\!}\mathscr {D}}_{\grave{\imath }_1^{+}}^{\upkappa ; {\uppsi }} \mathscr {A}(\texttt{t}). \end{aligned}$$

Apply system (8), to get

$$\begin{aligned} {{}^{\text {C}\!}\mathscr {D}}_{\grave{\imath }_1^{+}}^{\upkappa ; {\uppsi }}\mathfrak {L}(\texttt{t})&= \vartheta _1 \left\{ \upbeta _1\mathscr {S}\mathscr {I}+\upbeta _2\mathscr {S}\mathscr {A}-(\upupsilon +\upsigma )\mathscr {E}\right\} \\&\quad + \vartheta _2\left\{ \mathfrak {p}\upupsilon \mathscr {E}-(\upalpha +\upsigma +\updelta )\mathscr {I}\right\} + \vartheta _3 \left\{ (1-\mathfrak {p})\upupsilon \mathscr {E}-(\upgamma +\upsigma )\mathscr {A} \right\} \\&= \left[ \vartheta _1\upbeta _2\dfrac{\varLambda }{\upsigma }-\vartheta _3(\upgamma +\upsigma )\right] \mathscr {A} +\left[ \vartheta _1\upbeta _1\frac{\varLambda }{\upsigma }-\vartheta _2(\upalpha +\upsigma +\updelta ) \right] \mathscr {I} +\big [\vartheta _3(1-\mathfrak {p})\upupsilon + \vartheta _2\mathfrak {p}\upupsilon - \vartheta _1 (\upupsilon +\upsigma )\big ]\mathscr {E}, \end{aligned}$$

where $\vartheta _1=(\upalpha +\upsigma +\updelta )(\upgamma +\upsigma )$, $\vartheta _2=\upbeta _1\frac{\varLambda }{\upsigma }(\upgamma +\upsigma )$ and $\vartheta _3 = \upbeta _2\frac{\varLambda }{\upsigma }(\upalpha +\upsigma +\updelta )$. Therefore,

$$\begin{aligned} {{}^{\text {C}\!}\mathscr {D}}_{\grave{\imath }_1^{+}}^{\upkappa ; {\uppsi }}\mathfrak {L}(\texttt{t})= (\upalpha +\upsigma +\updelta )(\upupsilon +\upsigma )(\upgamma +\upsigma )[\breve{\mathscr {R}}_0-1]\mathscr {E}. \end{aligned}$$

Thus, if $\breve{\mathscr {R}}_0 \le 1$, then ${{}^{\text {C}\!}\mathscr {D}}_{\grave{\imath }_1^{+}}^{\upkappa ; {\uppsi }}\mathfrak {L}(\texttt{t})\le 0$. So, the DFE point of (8) is globally asymptotically stable whenever $\breve{\mathscr {R}}_0 \le 1$. $\square$

### $\breve{\mathscr {R}}_0$ sensitivity analysis

To study the $\breve{\mathscr {R}}_0$ sensitivity, we find the derivatives of it in the following way:

$$\begin{aligned} \frac{\partial \breve{\mathscr {R}}_0 }{\partial \upbeta _1}&=\frac{\mathfrak {p}\upupsilon \varLambda }{\upsigma (\upupsilon +\upsigma )(\upalpha +\upsigma +\updelta )},\qquad \frac{\partial \breve{\mathscr {R}}_0 }{\partial \upbeta _2}=\frac{(1-\mathfrak {p})\upupsilon \varLambda }{\upsigma (\upupsilon +\upsigma )(\upgamma +\upsigma )},\\ \frac{\partial \breve{\mathscr {R}}_0 }{\partial \varLambda }&=\frac{\mathfrak {p}\upupsilon \upbeta _1}{\upsigma (\upupsilon +\upsigma )(\upalpha +\upsigma +\updelta )} +\frac{(1-\mathfrak {p})\upupsilon \upbeta _2}{\upsigma (\upupsilon +\upsigma )(\upgamma +\upsigma )},\\ \frac{\partial \breve{\mathscr {R}}_0 }{\partial \upupsilon }&=\frac{\mathfrak {p}\upbeta _1\varLambda \upsigma }{\upsigma (\upupsilon +\upsigma )^2(\upalpha +\upsigma +\updelta )} +\frac{(1-\mathfrak {p})\upbeta _2\varLambda \upsigma }{\upsigma (\upgamma +\upsigma )(\upupsilon +\upsigma )^2},\\ \frac{\partial \breve{\mathscr {R}}_0 }{\partial \upalpha }&=-\frac{\mathfrak {p} \upupsilon \upbeta _1\varLambda }{\upsigma (\upupsilon +\upsigma )(\upalpha +\upsigma +\updelta )^2},\qquad \frac{\partial \breve{\mathscr {R}}_0 }{\partial \updelta }=-\frac{\mathfrak {p}\upupsilon \upbeta _1 \varLambda }{\upsigma (\upupsilon +\upsigma )(\upalpha +\upsigma +\updelta )^2},\\ \frac{\partial \breve{\mathscr {R}}_0 }{\partial \upgamma }&=-\frac{(1-\mathfrak {p})\upupsilon \upbeta _2\varLambda }{\upsigma (\upupsilon +\upsigma )(\upgamma +\upsigma )^2},\\ \frac{\partial \breve{\mathscr {R}}_0 }{\partial \upsigma }&=-\frac{(\upupsilon \upalpha +2\upsigma \upupsilon +2\upsigma \upalpha +\upupsilon \updelta +3\upsigma ^2+2\upsigma \updelta )(\mathfrak {p}\upupsilon \upbeta _1 \varLambda )}{(\upsigma (\upupsilon +\upsigma )(\upalpha +\upsigma +\updelta ))^2} \\&\quad -\frac{(\upupsilon \upgamma +2\upsigma \upupsilon +2\upsigma \upgamma +3\upsigma ^2)(1-\mathfrak {p}) \upupsilon \upbeta _2\varLambda }{(\upsigma (\upupsilon +\upsigma )(\upgamma +\upsigma ))^2}.  \end{aligned}$$

Since all the parameters are positive, so

$$\begin{aligned} \frac{\partial \breve{\mathscr {R}}_0}{\partial \upbeta _1}>0,\ \frac{\partial \breve{\mathscr {R}}_0}{\partial \upbeta _2}>0, \ \frac{\partial \breve{\mathscr {R}}_0}{\partial \varLambda }>0, \ \frac{\partial \breve{\mathscr {R}}_0}{\partial \upupsilon }>0, \ \frac{\partial \breve{\mathscr {R}}_0}{\partial \upalpha }<0, \ \frac{\partial \breve{\mathscr {R}}_0}{\partial \updelta }<0, \ \frac{\partial \breve{\mathscr {R}}_0}{\partial \upgamma }<0, \ \frac{\partial \breve{\mathscr {R}}_0}{\partial \upsigma }<0. \end{aligned}$$

In this way, $\breve{\mathscr {R}}_0$ is increasing with $\upbeta _1$, $\upbeta _2$, $\varLambda$, $\upupsilon$, but it is decreasing with $\upalpha$, $\updelta$, $\upgamma$, $\upsigma$.

## Existence and uniqueness of solution

It will be shown here that the system with the IC has a unique solution. To begin with, we compose system (8) as takes after:

$$\begin{aligned} &\uptheta ^{\upkappa -1}{{}^{\text {C}\!}\mathscr {D}}_{\grave{\imath }_1^{+}}^{\upkappa ; {\uppsi }}\mathscr {S}=\mathscr {H}_1 \left( \texttt{t},\mathscr {S} \right) ,\qquad \uptheta ^{\upkappa -1}{{}^{\text {C}\!}\mathscr {D}}_{\grave{\imath }_1^{+}}^{\upkappa ; {\uppsi }}\mathscr {E}=\mathscr {H}_2(\texttt{t},\mathscr {E}),\qquad \uptheta ^{\upkappa -1}{{}^{\text {C}\!}\mathscr {D}}_{\grave{\imath }_1^{+}}^{\upkappa ; {\uppsi }}\mathscr {I} = \mathscr {H}_3(\texttt{t},\mathscr {I}),\\&\uptheta ^{\upkappa -1}{{}^{\text {C}\!}\mathscr {D}}_{\grave{\imath }_1^{+}}^{\upkappa ; {\uppsi }}\mathscr {A}=\mathscr {H}_4(\texttt{t},\mathscr {A}),\qquad \uptheta ^{\upkappa -1}{{}^{\text {C}\!}\mathscr {D}}_{\grave{\imath }_1^{+}}^{\upkappa ; {\uppsi }}\mathscr {R}=\mathscr {H}_5(\texttt{t},\mathscr {R}, \end{aligned}$$

where

$$\begin{aligned} \mathscr {H}_1(\texttt{t},\mathscr {S})&= \varLambda - \upbeta _1 \mathscr {S}(\texttt{t})\mathscr {I} - \upbeta _2 \mathscr {S} \mathscr {A} - \upsigma \, \mathscr {S} + \varsigma \mathscr {R},\\ \mathscr {H}_2(\texttt{t},\mathscr {E})&= \upbeta _1 \mathscr {S}( \texttt{t})\mathscr {I} + \upbeta _2 \mathscr {S} \mathscr {A} - (\upupsilon + \upsigma ) \mathscr {E},\\ \mathscr {H}_3(\texttt{t}, \mathscr {I})&= \mathfrak {p} \upupsilon \mathscr {E} - (\upalpha + \upsigma +\updelta ) \mathscr {I},\\ \mathscr {H}_4(\texttt{t},\mathscr {A})&= ( 1- \mathfrak {p} ) \upupsilon \mathscr {E} - (\upgamma + \upsigma ) \mathscr {A},\\ \mathscr {H}_5(\texttt{t},\mathscr {S})&= \upalpha \mathscr {R} + \upgamma \mathscr {A} - (\upsigma + \varsigma ) \mathscr {R}. \end{aligned}$$

By taking integral, we have

10 $$\begin{aligned} {\left\{ \begin{array}{ll} \displaystyle \mathscr {S}(\texttt{t})-\mathscr {S}(0) = \uptheta ^{1-\upkappa } \int _{0}^{\texttt{t}} \frac{\uppsi ^\prime (\xi )}{(\tilde{\uppsi }_{\xi }(\texttt{t}) )^{1-\upkappa }} \frac{\mathscr {H}_1(\xi ,\mathscr {S})}{\Gamma (\upkappa )} \, \text {d}\xi ,\\ \displaystyle \mathscr {E}(\texttt{t}) - \mathscr {E}(0) = \uptheta ^{1-\upkappa } \int _{0}^{\texttt{t}} \frac{\uppsi ^\prime (\xi )}{ (\tilde{\uppsi }_{\xi }(\texttt{t}))^{1-\upkappa }} \frac{\mathscr {H}_2(\xi ,\mathscr {E})}{\Gamma (\upkappa )} \, \text {d}\xi ,\\ \displaystyle \mathscr {I}(\texttt{t})-\mathscr {I}(0) = \uptheta ^{1-\upkappa } \int _{0}^{\texttt{t}} \frac{\uppsi ^\prime (\xi )}{(\tilde{\uppsi }_{\xi }(\texttt{t}) )^{1-\upkappa }} \frac{\mathscr {H}_3(\xi ,\mathscr {I})}{\Gamma (\upkappa )} \, \text {d}\xi ,\\ \displaystyle \mathscr {A} ( \texttt{t})-\mathscr {A}(0)=\uptheta ^{1-\upkappa } \int _{0}^{\texttt{t}} \frac{\uppsi ^\prime (\xi )}{ (\tilde{\uppsi }_{\xi }(\texttt{t}))^{1 - \upkappa }}\frac{\mathscr {H}_4(\xi ,\mathscr {A})}{\Gamma (\upkappa )} \, \text {d}\xi ,\\ \displaystyle \mathscr {R}(\texttt{t})-\mathscr {R}(0)= \uptheta ^{1-\upkappa } \int _{0}^{\texttt{t}} \frac{\uppsi ^\prime (\xi )}{(\tilde{\uppsi }_{\xi }(\texttt{t}))^{1-\upkappa }} \frac{\mathscr {H}_5(\xi ,\mathscr {R})}{\Gamma (\upkappa )} \, \text {d}\xi . \end{array}\right. } \end{aligned}$$

### Theorem 4.1

*The kernel of the model will satisfy the Lipschitz condition* (LC) *if the disparity* $0 \le \mathfrak {g}_\imath <1$, $1\le \imath \le 5$, *hold, where* $\mathfrak {g}_1=\upbeta _1\mathfrak {r}_1+\upbeta _2\mathfrak {r}_2+\upsigma$, $\mathfrak {g}_2=\upupsilon +\upsigma$, $\mathfrak {g}_3=\upalpha +\upsigma +\updelta$, $\mathfrak {g}_4=\upgamma +\upsigma$ and $\mathfrak {g}_5 = \upsigma +\varsigma$.

### Proof

We will show for the first part and do the same for the rest. Consider functions $\mathscr {S}(\texttt{t})$ and $\mathscr {S}_1(\texttt{t})$. Then,

$$\begin{aligned} \Vert \mathscr {H}_1(\texttt{t},\mathscr {S}) - \mathscr {H}_1(\texttt{t},\mathscr {S}_1)\Vert&=\Vert -(\upbeta _1\mathscr {I} + \upbeta _2\mathscr {A} ) (\mathscr {S} - \mathscr {S}_1) - \upsigma (\mathscr {S}-\mathscr {S}_1 ) \Vert \\&\le \Vert \upbeta _1\mathscr {I} + \upbeta _2\mathscr {A} \Vert \, \Vert \mathscr {S} - \mathscr {S}_1\Vert + \upsigma \Vert \mathscr {S} - \mathscr {S}_1 \Vert \\&\le (\upbeta _1\Vert \mathscr {I} \Vert + \upbeta _2 \Vert \mathscr {A} \Vert + \upsigma ) \Vert \mathscr {S} - \mathscr {S}_1\Vert \\&\le ( \upbeta _1 \mathfrak {r}_1 + \upbeta _2\mathfrak {r}_2 + \upsigma ) \Vert \mathscr {S} - \mathscr {S}_1\Vert , \end{aligned}$$

where $\Vert \mathscr {I} \Vert \le \mathfrak {r}_1$, $\Vert \mathscr {A} \Vert \le \mathfrak {r}_2$. In a similar manner, we get

11 $$\begin{aligned}  \Vert \mathscr {H}_2(\texttt{t},\mathscr {E})-\mathscr {H}_2(\texttt{t},\mathscr {E}_1)\Vert \le \mathfrak {g}_2 \Vert \mathscr {E} - \mathscr {E}_1\Vert ,\qquad \Vert \mathscr {H}_3(\texttt{t}, \mathscr {I}) - \mathscr {H}_3 ( \texttt{t},\mathscr {I}_1) \Vert \le \mathfrak {g}_3 \Vert \mathscr {I} - \mathscr {I}_1\Vert ,\\ \Vert \mathscr {H}_4(\texttt{t},\mathscr {A})-\mathscr {H}_4(\texttt{t},\mathscr {A}_1 ) \Vert \le \mathfrak {g}_4 \Vert \mathscr {A} - \mathscr {A}_1 \Vert ,\qquad \Vert \mathscr {H}_5(\texttt{t}, \mathscr {R}) - \mathscr {H}_5 ( \texttt{t},\mathscr {R}_1)\Vert \le \mathfrak {g}_5 \Vert \mathscr {R} - \mathscr {R}_1 \Vert .  \end{aligned}$$

$$\square $$

Let’s look at some recursive versions of system (10),

$$\begin{aligned} \digamma _{1\texttt{n}}(\texttt{t})&= \mathscr {S}_\texttt{n} - \mathscr {S}_{\texttt{n}-1} = \frac{\uptheta ^{1-\upkappa }}{\Gamma (\upkappa )} \int _{0}^{\texttt{t}} \frac{\uppsi ^\prime (\xi )}{ (\tilde{\uppsi }_{\xi }(\texttt{t}) )^{ 1 - \upkappa }}( \mathscr {H}_1(\xi , \mathscr {S}_{ \texttt{n}-1}) - \mathscr {H}_1(\xi ,\mathscr {S}_{\texttt{n}-2}))\, \text {d}\xi ,\\ \digamma _{2\texttt{n}}(\texttt{t})&= \mathscr {E}_\texttt{n} - \mathscr {E}_{\texttt{n}-1} = \frac{\uptheta ^{ 1 - \upkappa }}{\Gamma (\upkappa )} \int _{0}^{\texttt{t}} \frac{\uppsi ^\prime (\xi )}{(\tilde{\uppsi }_{\xi }(\texttt{t}))^{ 1 - \upkappa }}( \mathscr {H}_2(\xi , \mathscr {E}_{\texttt{n}-1}) - \mathscr {H}_2(\xi ,\mathscr {E}_{\texttt{n}-2}))\, \text {d}\xi ,\\ \digamma _{3\texttt{n}}(\texttt{t})&= \mathscr {I}_\texttt{n} - \mathscr {I}_{\texttt{n}-1} = \frac{\uptheta ^{1-\upkappa }}{ \Gamma (\upkappa )} \int _{0}^{\texttt{t}} \frac{\uppsi ^\prime (\xi )}{(\tilde{\uppsi }_{\xi }(\texttt{t}))^{ 1 - \upkappa }}(\mathscr {H}_3( \xi ,\mathscr {I}_{ \texttt{n}-1})-\mathscr {H}_3(\xi ,\mathscr {I}_{\texttt{n}-2}))\, \text {d}\xi ,\\ \digamma _{4\texttt{n}}(\texttt{t})&=\mathscr {A}_\texttt{n} - \mathscr {A}_{\texttt{n}-1} = \frac{\uptheta ^{1-\upkappa }}{\Gamma (\upkappa )} \int _{0}^{\texttt{t}} \frac{\uppsi ^\prime (\xi )}{ (\tilde{\uppsi }_{\xi }(\texttt{t}) )^{ 1 - \upkappa }}(\mathscr {H}_4(\xi ,\mathscr {A}_{\texttt{n}-1})-\mathscr {H}_4(\xi ,\mathscr {A}_{\texttt{n}-2}))\, \text {d}\xi ,\\ \digamma _{5\texttt{n}}(\texttt{t})&=\mathscr {R}_\texttt{n} - \mathscr {R}_{\texttt{n}-1} = \frac{\uptheta ^{1-\upkappa }}{\Gamma (\upkappa )}\int _{0 }^{\texttt{t}} \frac{\uppsi ^\prime (\xi )}{(\tilde{\uppsi }_{\xi }(\texttt{t}))^{ 1 - \upkappa }}(\mathscr {H}_5(\xi ,\mathscr {R}_{\texttt{n}-1})-\mathscr {H}_5(\xi ,\mathscr {R}_{\texttt{n}-2}))\, \text {d}\xi , \end{aligned}$$

with the ICs $\mathscr {S}_0(\texttt{t})=\mathscr {S}(0)$, $\mathscr {E}_0(\texttt{t})=\mathscr {E}(0)$, $\mathscr {I}_0(\texttt{t})=\mathscr {I}(0)$, $\mathscr {A}_0(\texttt{t})=\mathscr {A}(0)$, and $\mathscr {R}_0(\texttt{t})=~\mathscr {R}(0)$. Hence,

$$\begin{aligned} \Vert \digamma _{1\texttt{n}}(\texttt{t})\Vert = \Vert \mathscr {S}_{\texttt{n}} - \mathscr {S}_{\texttt{n}-1} \Vert&=\left\| \frac{\uptheta ^{1-\upkappa }}{\Gamma (\upkappa )}\int _{0}^{ \texttt{t}} \frac{ \uppsi ^\prime (\xi )}{(\tilde{\uppsi }_{\xi }(\texttt{t}))^{1-\upkappa }}(\mathscr {H}_1(\xi ,\mathscr {S}_{\texttt{n}-1}) -\mathscr {H}_1(\xi ,\mathscr {S}_{\texttt{n}-2}))\, \text {d}\xi \right\| \\&\le \frac{ \uptheta ^{1-\upkappa }}{ \Gamma (\upkappa )}\int _{0}^{\texttt{t}} \left\| \frac{ \uppsi ^\prime (\xi )}{(\tilde{\uppsi }_{\xi }(\texttt{t}))^{1-\upkappa }}(\mathscr {H}_1(\xi , \mathscr {S}_{\texttt{n}-1})-\mathscr {H}_1(\xi ,\mathscr {S}_{\texttt{n}-2}))\right\| \, \text {d}\xi . \end{aligned}$$

With LC (11),

12 $$\begin{aligned} \Vert \digamma _{1\texttt{n}}(\texttt{t})\Vert \le \frac{\uptheta ^{1-\upkappa }}{\Gamma (\upkappa )} \mathfrak {g}_1\int _{0}^{\texttt{t}}\Vert \digamma _{1(\texttt{n}-1)}(\xi )\Vert \, \text {d}\xi . \end{aligned}$$

In a similar manner,

13 $$\begin{aligned} \Vert \digamma _{2\texttt{n}}\Vert&\le \frac{\uptheta ^{1-\upkappa }}{\Gamma (\upkappa )} \mathfrak {g}_2\int _{0}^{\texttt{t}}\Vert \digamma _{2(\texttt{n}-1)}(\xi )\Vert \, \text {d}\xi ,\qquad \Vert \digamma _{3\texttt{n}} \Vert \le \frac{\uptheta ^{1-\upkappa }}{\Gamma (\upkappa )}\mathfrak {g}_3\int _{0}^{\texttt{t}}\Vert \digamma _{3(\texttt{n}-1)}(\xi )\Vert \, \text {d}\xi ,\\ \Vert \digamma _{4\texttt{n}} \Vert&\le \frac{\uptheta ^{1-\upkappa }}{\Gamma (\upkappa )}\mathfrak {g}_4\int _{0}^{\texttt{t}}\Vert \digamma _{4(\texttt{n}-1)}(\xi )\Vert \, \text {d}\xi ,\qquad \Vert \digamma _{5\texttt{n}} \Vert \le \frac{\uptheta ^{1-\upkappa }}{\Gamma (\upkappa )}\mathfrak {g}_5\int _{0}^{\texttt{t}}\Vert \digamma _{5(\texttt{n}-1)}(\xi )\Vert \, \text {d}\xi .  \end{aligned}$$

Thus,

$$\begin{aligned} \mathscr {S}_\texttt{n}(\texttt{t}) = \sum _{\mathfrak {i}=1}^{\texttt{n}}\digamma _{1\mathfrak {i}}(\texttt{t}), \mathscr {E}_\texttt{n}(\texttt{t})=\sum _{\mathfrak {i}=1}^{\texttt{n}}\digamma _{2\mathfrak {i}}(\texttt{t}), \mathscr {I}_\texttt{n}(\texttt{t})=\sum _{\mathfrak {i}=1}^{\texttt{n}}\digamma _{3\mathfrak {i}}(\texttt{t}), \mathscr {A}_\texttt{n}(\texttt{t})=\sum _{\mathfrak {i}=1}^{\texttt{n}}\digamma _{4\mathfrak {i}}(\texttt{t}), \mathscr {R}_\texttt{n}(\texttt{t})=\sum _{\mathfrak {i}=1}^{\texttt{n}}\digamma _{5\mathfrak {i}}(\texttt{t}). \end{aligned}$$

### Theorem 4.2

*If there exists* $\texttt{t}_1$ *such that* $\frac{1}{ \Gamma (\upkappa )} \uptheta ^{1-\upkappa } \texttt{t}_1 \mathfrak {g}_{\ell }<1$, *then a the solution of the system of fractional* $\mathscr {C}\mathscr {O}\mathscr {V}\mathscr {I}\mathscr {D}$-${\textbf {19}}$ $\mathscr {S}\mathscr {E}\mathscr {I}\mathscr {A}\mathscr {R}\mathscr {S}$ *model* (8) *exists.*

### Proof

From the recessive method and Eqs. (12) and (13) we conclude that

$$\begin{aligned}  \Vert \digamma _{1\texttt{n}}\Vert&\le \Vert \mathscr {S}_\texttt{n}(0)\Vert \left[ \frac{\uptheta ^{1-\upkappa }}{\Gamma (\upkappa )}\mathfrak {g}_1\texttt{t}\right] ^\texttt{n},\quad \Vert \digamma _{2\texttt{n}}\Vert \le \Vert \mathscr {E}_\texttt{n}(0)\Vert \left[ \frac{\uptheta ^{1-\upkappa }}{\Gamma (\upkappa )}\mathfrak {g}_2\texttt{t}\right] ^\texttt{n},\quad \Vert \digamma _{3\texttt{n}}\Vert \le \Vert \mathscr {I}_\texttt{n}(0)\Vert \left[ \frac{\uptheta ^{1-\upkappa }}{\Gamma (\upkappa )}\mathfrak {g}_3\texttt{t}\right] ^\texttt{n},\\ \Vert \digamma _{4\texttt{n}}\Vert&\le \Vert \mathscr {A}_\texttt{n}(0)\Vert \left[ \frac{\uptheta ^{1-\upkappa }}{\Gamma (\upkappa )}\mathfrak {g}_4\texttt{t}\right] ^\texttt{n},\quad \Vert \digamma _{5\texttt{n}}\Vert \le \Vert \mathscr {R}_\texttt{n}(0)\Vert \left[ \frac{\uptheta ^{1-\upkappa }}{\Gamma (\upkappa )}\mathfrak {g}_5\texttt{t}\right] ^\texttt{n}. \end{aligned}$$

Hence, the system  (8) has a solution and also it is continuous. Now, we prove that the upper functions fabricate solution for model (10). Let $\mathscr {S}(\texttt{t})-\mathscr {S}(0)$ $=\mathscr {S}_\texttt{n} - G_{1\texttt{n}}$, $\mathscr {E}(\texttt{t})-\mathscr {E}(0)$ $= \mathscr {E}_\texttt{n} - G_{2\texttt{n}}$, $\mathscr {I}(\texttt{t}) - \mathscr {I}(0)$ $=\mathscr {I}_\texttt{n} - G_{3\texttt{n}}$, $\mathscr {A}(\texttt{t})-\mathscr {A}(0)= \mathscr {A}_\texttt{n}- G_{4\texttt{n}}$, $\mathscr {R}(\texttt{t}) - \mathscr {R}(0)$ $=\mathscr {R}_\texttt{n} - G_{5\texttt{n}}$. Thus,

$$\begin{aligned}  \Vert G_{1\texttt{n}}\Vert&=\left\| \frac{\uptheta ^{1-\upkappa }}{\Gamma (\upkappa )}\int _{0}^{\texttt{t}}(\mathscr {H}_1(\xi ,\mathscr {S}) -\mathscr {H}_1(\xi ,\mathscr {S}_{\texttt{n}-1})\, \text {d}\xi \right\| \\&\le \frac{\uptheta ^{1-\upkappa }}{\Gamma (\upkappa )}\int _{0}^{\texttt{t}}\Vert (\mathscr {H}_1(\xi ,\mathscr {S}) -\mathscr {H}_1(\xi ,\mathscr {S}_{\texttt{n}-1})\Vert \, \text {d}\xi \le \frac{\uptheta ^{1-\upkappa }}{\Gamma (\upkappa )}\mathfrak {g}_1\Vert \mathscr {S}-\mathscr {S}_{\texttt{n}-1}\Vert \texttt{t}. \end{aligned}$$

With iterate the procedure, we have, at $\texttt{t}_1$,

$$\begin{aligned} \Vert G_{1\texttt{n}}(\texttt{t})\Vert \le \left[ \frac{\uptheta ^{1-\upkappa }}{\Gamma (\upkappa )} \texttt{t}_1\right] ^{\texttt{n}+1}\mathfrak {g}_1^{\texttt{n}+1}c. \end{aligned}$$

When we make the value of $\texttt{n}$ go towards infinity, the upper equation gives us a limit, $\Vert G_{ 1 \texttt{n}}\Vert \rightarrow 0$. In a similar manner, one can check that $\Vert G_{\ell \texttt{n}} \Vert \rightarrow 0$, $\ell = 2, 3, 4, 5$. This completes the proof. $\square$

### Theorem 4.3

*Suppose that* $1-\frac{\uptheta ^{1-\upkappa }}{\Gamma (\upkappa )}\mathfrak {g}_1\texttt{t} >0.$ *Then, the solution of* $\mathscr {S}\mathscr {E}\mathscr {I}\mathscr {A}\mathscr {R}\mathscr {S}$ *model* (8) *is unique.*

### Proof

To show that the solution is one, we assume that there is another solution called $\mathscr {S}_1$, $\mathscr {E}_1$, $\mathscr {I}_1$, $\mathscr {A}_1$, and $\mathscr {R}_1$. Then,

$$\begin{aligned} \mathscr {S}(\texttt{t})-\mathscr {S}_1(\texttt{t})=\frac{\uptheta ^{1-\upkappa }}{\Gamma (\upkappa )} \int _{0}^{\texttt{t}}(\mathscr {H}_1(\xi ,\mathscr {S})-\mathscr {H}_1(\xi ,\mathscr {S}_{1}))\, \text {d}\xi . \end{aligned}$$

Therefore,

$$\begin{aligned} \Vert \mathscr {S}-\mathscr {S}_1\Vert \le \frac{\uptheta ^{1-\upkappa }}{\Gamma (\upkappa )}\int _{0}^{\texttt{t}}\Vert \mathscr {H}_1(\xi ,\mathscr {S}) -\mathscr {H}_1(\xi ,\mathscr {S}_{1})\Vert \, \text {d}\xi . \end{aligned}$$

It follows from (11) that $\Vert \mathscr {S} - \mathscr {S}_1 \Vert \le \frac{1}{ \Gamma ( \upkappa )} \, \uptheta ^{1-\upkappa } \, \mathfrak {g}_1\texttt{t}\Vert \mathscr {S}(\texttt{t})-\mathscr {S}_1(\texttt{t})\Vert .$ Hence,

$$\begin{aligned} \Vert \mathscr {S}(\texttt{t})-\mathscr {S}_1(\texttt{t})\Vert \left( 1-\frac{\uptheta ^{1-\upkappa }}{\Gamma (\upkappa )}\mathfrak {g}_1\texttt{t}\right) \le 0. \end{aligned}$$

Then, $\Vert \mathscr {S} - \mathscr {S}_1 \Vert =0$. Therefore, $\mathscr {S}=\mathscr {S}_1$. In the same way, we are able to display the same parity for $\mathscr {E}, \mathscr {I}, \mathscr {A}, \mathscr {R}$. $\square$

## Numerical results

Utilizing the FEP for $\uppsi$-CFD, approximate solutions for the fractional-order $\mathscr {C}$-19, $\mathscr {S}\mathscr {E}\mathscr {I}\mathscr {A}\mathscr {R}\mathscr {S}$ model will be provided (see^43^). Simulations to foreknow the $\mathscr {C}$-19 transmission within the world will also be provided.

### Numerical procedure

Let’s think about system (8) in a shorter and simpler way as:

14 $$\begin{aligned} \uptheta ^{\upkappa -1}{{}^{\text {C}\!}\mathscr {D}}_{\grave{\imath }_1^{+}}^{\upkappa ; {\uppsi }} \uplambda = \upomega (\texttt{t}, \uplambda ), \qquad \uplambda (0)=\uplambda _0=(\mathscr {S}_0,\mathscr {E}_0,\mathscr {I}_0,\mathscr {A}_0,\mathscr {R}_0),\ 0 \le \texttt{t}\le \mathscr {T}<\infty , \end{aligned}$$

where $\uplambda =(\mathscr {S},\mathscr {E},\mathscr {I},\mathscr {A},\mathscr {R})\in  {R}_+^5$ and $\upomega (\texttt{t})\in ~ {R}$ is a continuous vector function satisfying LC

$$\begin{aligned} \Vert \upomega (\uplambda _1(\texttt{t}))-\upomega (\uplambda _2(\texttt{t}))\Vert \le c\Vert \uplambda _1) - \uplambda _2\Vert ,\qquad c>0. \end{aligned}$$

Exerting a fractional integral operator matching to the $\uppsi$-CFD to Eq. (14), we get

$$\begin{aligned} \uplambda \big |_{\texttt{t}} = \uptheta ^{1-\upkappa } \Big [ \uplambda _0 + \mathscr {I}^{\upkappa ; {\uppsi }}\upomega (\uplambda )\big |_{\texttt{t}} \Big ], \qquad 0 \le \texttt{t}\le \mathscr {T}< \infty . \end{aligned}$$

Set $\mathfrak {m}=\frac{\mathscr {T}-0}{\mathscr {N}}$, $\mathscr {N}\in \mathbb {N}$ and $\texttt{t}_{ \texttt{n}}=\texttt{n}\mathfrak {m}$, $\texttt{n}= 0, 1, 2,\dots ,\mathscr {N}$ for $0 \le \texttt{t}\le \mathscr {T}$. Let $\uplambda _{\texttt{n}}=\uplambda \big |_{\texttt{t}_{\texttt{n}}}$. Utilizing the FEP, see^43^, we obtain

$$\begin{aligned} \uplambda _{\texttt{n}+1}=\uptheta ^{1-\upkappa }\left[ \uplambda _0+\frac{\mathfrak {m}^{\upkappa }}{\Gamma (\upkappa +1)}\sum _{\mathfrak {i}=0}^{\texttt{n}}\texttt{u}_{\texttt{n}+1},_\mathfrak {i} \upomega (\texttt{t}_\mathfrak {i},\uplambda _\mathfrak {i})\right] ,\qquad \mathfrak {i}\in \{0\}\cup \mathbb {N}, \,0\le \mathfrak {i} \le =\mathscr {N}-1, \end{aligned}$$

where $\texttt{u}_{\texttt{n}+1},_\mathfrak {i}=(\texttt{n}+1-\mathfrak {i})^{\upkappa }-(\texttt{n}-\mathfrak {i})^{\upkappa }$, $\mathfrak {i}=0, 1, 2,\dots ,\texttt{n}$. The researchers have proven that the obtained scheme is stable in their work^43^, Theorem (3.1). As a result, the approximate solution is expressed by

$$\begin{aligned} \mathscr {S}_{\texttt{n}+1}&=\uptheta ^{1-\upkappa }\left[ \mathscr {S}_0+\frac{\mathfrak {m}^{\upkappa }}{\Gamma (\upkappa +1)} \sum _{\mathfrak {i}=0}^{\texttt{n}}\texttt{u}_{\texttt{n}+1},_\mathfrak {i}\mathscr {X}_1(\texttt{t}_\mathfrak {i},\uplambda _\mathfrak {i})\right] ,\\ \mathscr {E}_{\texttt{n}+1}&=\uptheta ^{1-\upkappa }\left[ \mathscr {E}_0+\frac{\mathfrak {m}^{\upkappa }}{\Gamma (\upkappa +1)} \sum _{\mathfrak {i}=0}^{\texttt{n}}\texttt{u}_{\texttt{n}+1},_\mathfrak {i}\mathscr {X}_2(\texttt{t}_\mathfrak {i},\uplambda _\mathfrak {i})\right] ,\\ \mathscr {I}_{\texttt{n}+1}&=\uptheta ^{1-\upkappa }\left[ \mathscr {I}_0+\frac{\mathfrak {m}^{\upkappa }}{\Gamma (\upkappa +1)} \sum _{\mathfrak {i}=0}^{\texttt{n}}\texttt{u}_{\texttt{n}+1},_\mathfrak {i}\mathscr {X}_3(\texttt{t}_\mathfrak {i},\uplambda _\mathfrak {i})\right] ,\\ \mathscr {A}_{\texttt{n}+1}&= \uptheta ^{1-\upkappa }\left[ \mathscr {A}_0+\frac{\mathfrak {m}^{\upkappa }}{\Gamma (\upkappa +1)} \sum _{\mathfrak {i}=0}^{\texttt{n}}\texttt{u}_{\texttt{n}+1},_\mathfrak {i}\mathscr {X}_4(\texttt{t}_\mathfrak {i},\uplambda _\mathfrak {i})\right] , \\ \mathscr {R}_{\texttt{n}+1}&=\uptheta ^{1-\upkappa }\left[ \mathscr {R}_0+\frac{\mathfrak {m}^{\upkappa }}{\Gamma (\upkappa +1)} \sum _{\mathfrak {i}=0}^{\texttt{n}}\texttt{u}_{\texttt{n}+1},_\mathfrak {i}\mathscr {X}_5(\texttt{t}_\mathfrak {i},\uplambda _\mathfrak {i})\right] , \end{aligned}$$

where

$$\begin{aligned} \mathscr {X}_1(\texttt{t}, \uplambda )&= \varLambda - \upbeta _1\mathscr {I} \mathscr {S} - \upbeta _2\mathscr {A} \mathscr {S}- \upsigma \mathscr {S} + \varsigma \mathscr {R},\qquad \mathscr {X}_2 (\texttt{t}, \uplambda ) = \upbeta _1\mathscr {I}\mathscr {S} + \upbeta _2\mathscr {A}\mathscr {S}- (\upupsilon +\upsigma ) \mathscr {E}, \\ \mathscr {X}_3 ( \texttt{t}, \uplambda )&= \mathfrak {p} \upupsilon \mathscr {E}- (\upalpha +\upsigma +\updelta )\mathscr {I},\quad \mathscr {X}_4(\texttt{t}, \uplambda ) = (1 - \mathfrak {p}) \upupsilon \mathscr {E} - (\upgamma +\upsigma ) \mathscr {A}, \quad \mathscr {X}_5(\texttt{t}, \uplambda ) = \upalpha \mathscr {I} +\upgamma \mathscr {A} - (\upsigma + \varsigma ) \mathscr {R}. \end{aligned}$$

### The model’s numerical simulations and interpretation

Using MATLAB, model (8) will be simulated for the world’s data. For simulation, the value of the parameters should be first determined. The birth and death rate for the world in 2022 were 17.668 births and 7.678 per 1000 individuals, respectively. The world’s population on 15 June was $\aleph = 7914981120$, so

$$\begin{aligned} \varLambda = \frac{1}{365}\, \texttt{n} \aleph =383128.455, \qquad \upsigma = \frac{7.678}{365\times 1000}=2.10356\times 10^{-5}, \end{aligned}$$

and we choose $\uptheta = 0.99$. Since $\aleph (0) = \mathscr {S}(0) + \mathscr {E}(0) + \mathscr {I}(0) + \mathscr {A}(0) + \mathscr {R}(0),$ and on 15 June $\mathscr {I}(0) = 15\ 315\ 220$, then we can suppose

$$\begin{aligned} \mathscr {E}(0)=20\ 000\ 000,\quad \mathscr {A}(0) =10\ 000\ 000, \quad \mathscr {R}(0)=0,\quad \mathscr {S}(0)=7\ 869\ 665\ 900. \end{aligned}$$

In addition, we consider the number of infection cases in the world in the period of time $\breve{T}$, 15 June to 4 August 2022, so that any part is three day. The parameters values of model (8) are available in Table 2. In this simulation, the EP is

$$\begin{aligned} \mathscr {E}_1=\left( \mathscr {S}^{\star},\ \mathscr {E}^{\star}, \ \mathscr {I}^{\star},\mathscr {A}^{\star}, \ \mathscr {R}^{\star } \right) = \Big (7.3\times 10^{9}, \ 1.9\times 10^{7},\ 1.47\times 10^{7},\ 1.43\times 10^{7},\ 5.5\times 10^{8}\Big ). \end{aligned}$$

**Table 2:** Details of the model parameters and their numerical value.

Parameter	Value	Ref
$\varLambda$	383128.455	$\text {Estimated}$
$\upsigma$	$2.10356\times 10^{-5}$	$\text {Estimated}$
$\upbeta _1$	$9.41\times 10^{-11}$	$\text {Fitted}$
$\upbeta _2$	$1\times 10^{-11}$	$\text {Fitted}$
$\varsigma$	0.02	$\text {Fitted}$
$\upalpha$	0.365	$\text {Fitted}$
$\upgamma$	0.3890	$\text {Fitted}$
$\upupsilon$	0.6	$\text {Fitted}$
$\updelta$	0.015	See^44,45^
$\mathfrak {p}$	0.5	See^46^

Problem (8) will be examined in three cases for $\uppsi (\texttt{t})$ as $\uppsi _1(\texttt{t}) = \texttt{t}$ (Caputo derivative); $\uppsi _2(\texttt{t}) = \ln \texttt{t}$ (Caputo–Hadamard derivative); $\uppsi _3(\texttt{t}) = \sqrt{\texttt{t}}$ (Katugampola derivative).

**Case I.** Let $\uppsi _1(\texttt{t}) = \texttt{t}$ (Caputo derivative).

**Figure 4:**
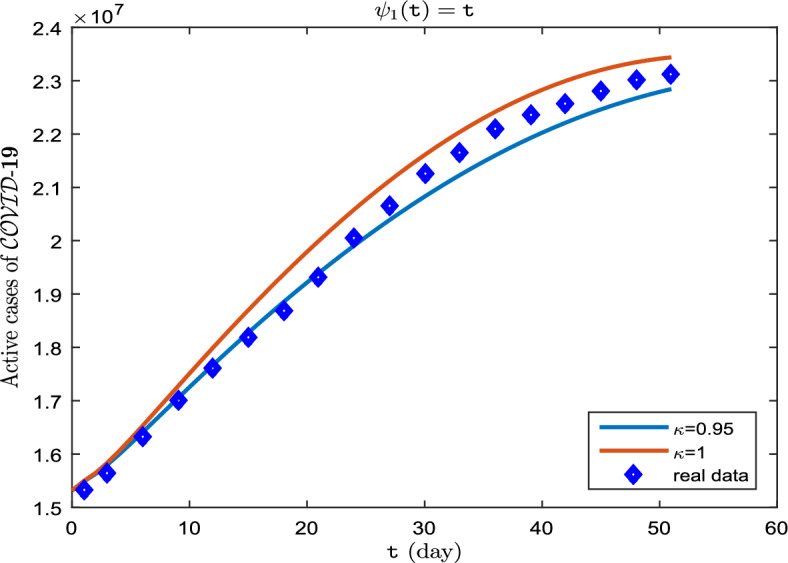
Active cases of $\mathscr {C}\mathscr {O}\mathscr {V}\mathscr {I}\mathscr {D}$-${\textbf {19}}$ on period $\breve{T}$.

**Figure 5:**
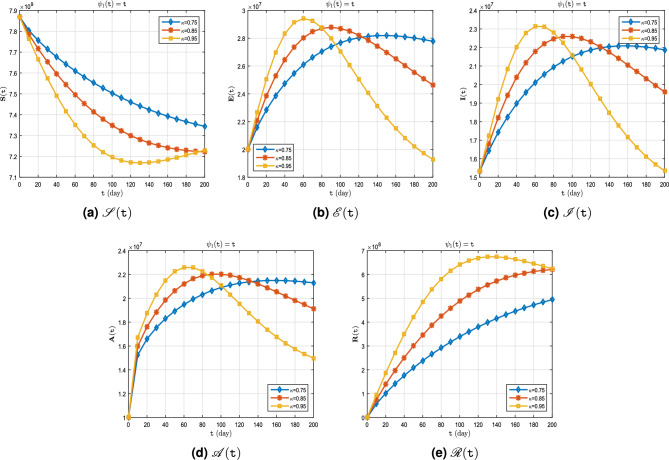
Dynamics of $\mathscr {S}(\texttt{t})$, $\mathscr {E}(\texttt{t})$, $\mathscr {I}(\texttt{t})$, $\mathscr {A}(\texttt{t})$ and $\mathscr {R}(\texttt{t})$ whenever $\uppsi _1(\texttt{t})= \texttt{t}$ and different fractional order $\upkappa = 0.75, 0.85, 0.95$.

The real data for infected cases, as well as the results of model (8) for $\upkappa \in \{0.95, 1\}$ on period $\breve{T}$ can be seen in Fig. 4. Also, in Fig. 5, it is predicted how each of the classes $\mathscr {S}$, $\mathscr {E}$, $\mathscr {I}$, $\mathscr {A}$ and $\mathscr {R}$ will change with $\upkappa \in \{ 0.75, 0.85, 0.95\}$. In addition, Tables 3, 4 and 5 show these results. An important point in this case, over the course of time, $\mathscr {S}$ decreases as the order of the derivative $\kappa$ approaches one (Fig. 5a), and even $\mathscr {E}$, $\mathscr {I}$, $\mathscr {A}$ (Fig. 5b–d) has a faster downward growth. But this is not the case for $\mathscr {R}$. In fact, as the order of the derivative $\kappa$ approaches one, the value of $\mathscr {R}$ increases more (Fig. 5e).

**Table 3:** Obtained results of $\mathscr {S}(\texttt{t})$, $\mathscr {E}(\texttt{t})$, $\mathscr {I}(\texttt{t})$, $\mathscr {A}(\texttt{t})$ and $\mathscr {R}(\texttt{t})$ whenever $\uppsi _1(\texttt{t})= \texttt{t}$ and fractional order $\upkappa = 0.75$.

$\texttt{t}$	$\uppsi _1(\texttt{t})= \texttt{t}, \quad {\upkappa =0.75}$				
	$\mathscr {S}$	$\mathscr {E}$	$\mathscr {I}$	$\mathscr {A}$	$\mathscr {R}$
0	7869665900	20000000	15315220	10000000	0
1	7860830016	20094962	15448671	11565115	7032637
2	7853033854	20290342	15549178	12506680	13581346
3	7845872620	20479004	15657315	13161083	19781136
4	7839154660	20658141	15769680	13650343	25707613
$\vdots$	$\vdots$	$\vdots$	$\vdots$	$\vdots$	$\vdots$
100	7503275503	27677663	21510744	20904263	339058082
101	7500987972	27701550	21533292	20927122	341244555
102	7498717194	27724779	21555308	20949446	343416005
103	7496463071	27747355	21576799	20971240	345572511
$\vdots$	$\vdots$	$\vdots$	$\vdots$	$\vdots$	$\vdots$
197	7347317561	27815672	21898637	21324880	491227069
198	7346286566	27802190	21889933	21316601	492263235
199	7345265086	27788540	21881077	21308171	493290454
200	7344253058	27774724	21872072	21299594	494308780

**Table 4:** Obtained results of $\mathscr {S}(\texttt{t})$, $\mathscr {E}(\texttt{t})$, $\mathscr {I}(\texttt{t})$, $\mathscr {A}(\texttt{t})$ and $\mathscr {R}(\texttt{t})$ whenever $\uppsi _1(\texttt{t})= \texttt{t}$ and fractional order $\upkappa = 0.85$.

$\texttt{t}$	$\uppsi _1(\texttt{t})= \texttt{t}, \quad {\upkappa =0.85}$				
	$\mathscr {S}$	$\mathscr {E}$	$\mathscr {I}$	$\mathscr {A}$	$\mathscr {R}$
0	7869665900	20000000	15315220	10000000	0
1	7859557659	20108637	15467888	11790490	8045329
2	7850149845	20355755	15587414	12881883	15982890
3	7841211719	20596005	15725920	13636203	23774862
4	7832614633	20836255	15875080	14192570	31420412
$\vdots$	$\vdots$	$\vdots$	$\vdots$	$\vdots$	$\vdots$
100	7348728654	28713862	22593761	22030108	488094733
101	7345923533	28700391	22588601	22025325	490868404
102	7343158332	28685723	22582423	22019541	493605516
103	7340432830	28669884	22575245	22012776	496306232
$\vdots$	$\vdots$	$\vdots$	$\vdots$	$\vdots$	$\vdots$
197	7222306548	24765884	19707170	19224245	619927754
198	7222087677	24720665	19671602	19189597	620234627
199	7221882302	24675618	19636145	19155057	620527904
200	7221690205	24630744	19600802	19120629	620807792

**Table 5:** Obtained results of $\mathscr {S}(\texttt{t})$, $\mathscr {E}(\texttt{t})$, $\mathscr {I}(\texttt{t})$, $\mathscr {A}(\texttt{t})$ and $\mathscr {R}(\texttt{t})$ whenever $\uppsi _1(\texttt{t})= \texttt{t}$ and fractional order $\upkappa = 0.95$.

$\texttt{t}$	$\uppsi _1(\texttt{t})= \texttt{t}, \quad {\upkappa =0.95}$				
	$\mathscr {S}$	$\mathscr {E}$	$\mathscr {I}$	$\mathscr {A}$	$\mathscr {R}$
0	7869665900	20000000	15315220	10000000	0
1	7858338610	20121739	15486300	12006423	9015592
2	7847222227	20424519	15625861	13253405	18428079
3	7836298804	20722154	15799259	14110785	28006791
4	7825526394	21014264	15992365	14733065	37653177
$\vdots$	$\vdots$	$\vdots$	$\vdots$	$\vdots$	$\vdots$
100	7197975425	27155399	21649805	21064204	638927206
101	7195949537	27058574	21576523	20991841	641112531
102	7194007150	26960975	21502443	20918758	643217693
103	7192147110	26862672	21427623	20845012	645243665
$\vdots$	$\vdots$	$\vdots$	$\vdots$	$\vdots$	$\vdots$
197	7225901360	19409011	15442967	15078422	627237292
198	7227098207	19367671	15408510	15044826	626144098
199	7228293553	19327053	15374636	15011799	625050914
200	7229487083	19287152	15341342	14979336	623958063

**Case II.** Let $\uppsi _2(\texttt{t}) = \ln \texttt{t}$ (Caputo–Hadamard derivative).

**Figure 6:**
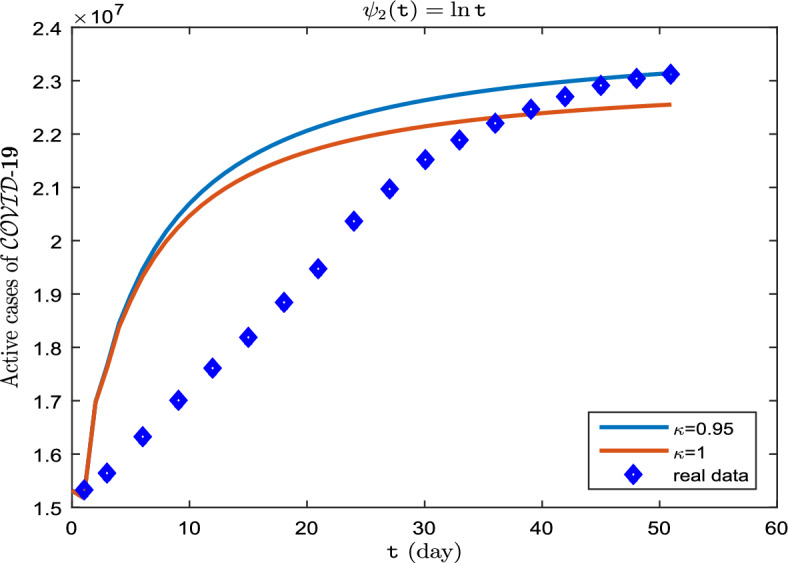
Active cases of $\mathscr {C}\mathscr {O}\mathscr {V}\mathscr {I}\mathscr {D}$-${\textbf {19}}$ on period $\breve{T}$.

**Figure 7:**
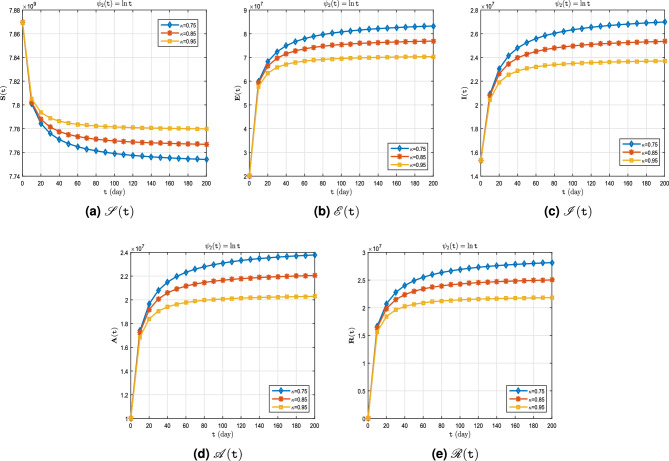
Dynamics of $\mathscr {S}(\texttt{t})$, $\mathscr {E}(\texttt{t})$, $\mathscr {I}(\texttt{t})$, $\mathscr {A}(\texttt{t})$ and $\mathscr {R}(\texttt{t})$ whenever $\uppsi _2(\texttt{t})= \ln \texttt{t}$ and different fractional order $\upkappa = 0.75, 0.85, 0.95$.

The real data for infected cases, as well as the results of model (8) for $\upkappa \in \{0.95, 1\}$ on period $\breve{T}$ can be seen in Fig. 6. Also, in Fig. 7, it is predicted how each of the classes $\mathscr {S}$, $\mathscr {E}$, $\mathscr {I}$, $\mathscr {A}$ and $\mathscr {R}$ will change with $\upkappa \in \{ 0.75, 0.85, 0.95\}$. In addition, Tables 6, 7 and 8 show these results. The existence of exponential changes in this case can be clearly seen in curves (Fig. 7a–e). What is received from the graphs and numerical results is indicative of the fact that in this case, $\uppsi _2(\texttt{t})= \ln \texttt{t}$, the natural logarithm function is not appropriate in the presented model (1) (Fig. 7).

**Table 6:** Obtained results of $\mathscr {S}(\texttt{t})$, $\mathscr {E}(\texttt{t})$, $\mathscr {I}(\texttt{t})$, $\mathscr {A}(\texttt{t})$ and $\mathscr {R}(\texttt{t})$ whenever $\uppsi _2(\texttt{t})= \ln \texttt{t}$ and fractional order $\upkappa = 0.75$.

$\texttt{t}$	$\uppsi _2(\texttt{t})= \ln \texttt{t}, \quad {\upkappa =0.75}$				
	$\mathscr {S}$	$\mathscr {E}$	$\mathscr {I}$	$\mathscr {A}$	$\mathscr {R}$
0	7869665900	20000000	15315220	10000000	0
1	7847025110	38005171	14084338	10373259	5488838
2	7838247767	40579825	15960764	12579909	7612759
3	7829582624	45695634	16559910	13420768	9714215
4	7823452484	48592485	17357955	14363787	11200151
$\vdots$	$\vdots$	$\vdots$	$\vdots$	$\vdots$	$\vdots$
100	7759049106	80773138	25001908	23099135	26917187
101	7758956822	80818724	25013128	23111712	26939874
102	7758866102	80863537	25024159	23124075	26962177
103	7758776903	80907598	25035003	23136231	26984107
$\vdots$	$\vdots$	$\vdots$	$\vdots$	$\vdots$	$\vdots$
197	7754097118	83219139	27112262	23773881	28135185
198	7754068964	83233046	27115466	23777716	28142113
199	7754041061	83246829	27118342	23781517	28148979
200	7754013408	83260489	27121293	23785284	28155784

**Table 7:** Obtained results of $\mathscr {S}(\texttt{t})$, $\mathscr {E}(\texttt{t})$, $\mathscr {I}(\texttt{t})$, $\mathscr {A}(\texttt{t})$ and $\mathscr {R}(\texttt{t})$ whenever $\uppsi _2(\texttt{t})= \ln \texttt{t}$ and fractional order $\upkappa = 0.85$.

$\texttt{t}$	$\uppsi _2(\texttt{t})= \ln \texttt{t}, \quad {\upkappa =0.85}$				
	$\mathscr {S}$	$\mathscr {E}$	$\mathscr {I}$	$\mathscr {A}$	$\mathscr {R}$
0	7869665900	20000000	15315220	10000000	0
1	7846396848	38504799	14050182	10383616	5641148
2	7837803252	40685450	16069035	12703258	7720281
3	7829043340	45995695	16609801	13478562	9845087
4	7823139350	48690923	17428856	14430944	11276111
$\vdots$	$\vdots$	$\vdots$	$\vdots$	$\vdots$	$\vdots$
100	7769720917	75446085	23740068	21655007	24300429
101	7769664754	75473938	23746841	21662644	24314205
102	7769609630	75501276	23753487	21670139	24327725
103	7769555515	75528113	23760012	21677498	24340998
$\vdots$	$\vdots$	$\vdots$	$\vdots$	$\vdots$	$\vdots$
197	7766856324	76875087	25406375	22046501	25007030
198	7766840626	76882804	25408124	22048613	25010844
199	7766825081	76890448	25410237	22050705	25014622
200	7766809684	76898018	25412135	22052777	25018364

**Table 8:** Obtained results of $\mathscr {S}(\texttt{t})$, $\mathscr {E}(\texttt{t})$, $\mathscr {I}(\texttt{t})$, $\mathscr {A}(\texttt{t})$ and $\mathscr {R}(\texttt{t})$ whenever $\uppsi _2(\texttt{t})= \ln \texttt{t}$ and fractional order $\upkappa = 0.95$.

$\texttt{t}$	$\uppsi _2(\texttt{t})= \ln \texttt{t}, \quad {\upkappa =0.95}$				
	$\mathscr {S}$	$\mathscr {E}$	$\mathscr {I}$	$\mathscr {A}$	$\mathscr {R}$
0	7869665900	20000000	15315220	10000000	0
1	7846249518	38621963	14042172	10386045	5676866
2	7837993793	40474811	16110976	12727784	7674030
3	7829455368	45757204	16587097	13427279	9745317
4	7823962932	48191831	17387067	14348039	11076428
$\vdots$	$\vdots$	$\vdots$	$\vdots$	$\vdots$	$\vdots$
100	7781453106	69561854	22368234	20072799	21429738
101	7781420755	69577988	22372085	20077183	21437653
102	7781389044	69593803	22375859	20081479	21445411
103	7781357955	69609308	22379559	20085691	21453017
$\vdots$	$\vdots$	$\vdots$	$\vdots$	$\vdots$	$\vdots$
197	7779855048	70359477	23804956	20289171	21820722
198	7779846797	70363600	23805932	20290287	21822740
199	7779838629	70367681	23806899	20291392	21824739
200	7779830544	70371721	23807856	20292486	21826717

**Case III.** Let $\uppsi _3(\texttt{t}) = \sqrt{\texttt{t}}$ (Katugampola derivative).

**Figure 8:**
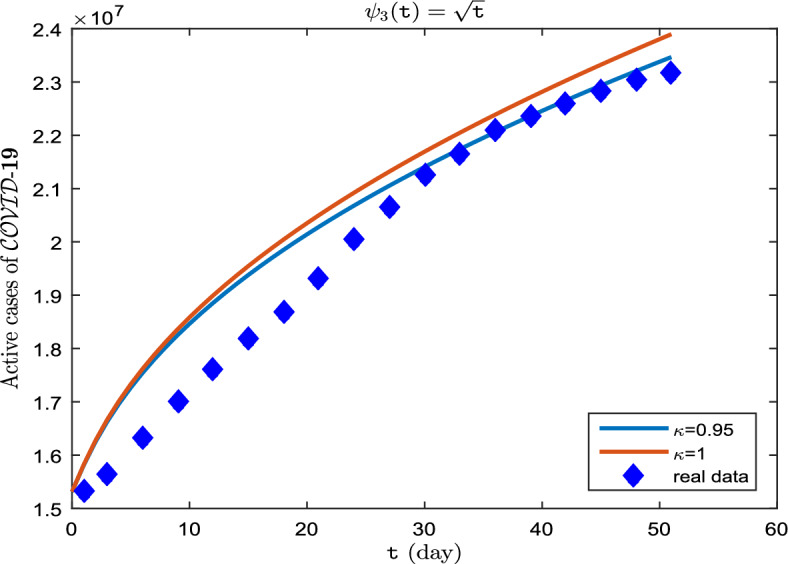
Active cases of $\mathscr {C}\mathscr {O}\mathscr {V}\mathscr {I}\mathscr {D}$-${\textbf {19}}$ on period $\breve{T}$.

**Figure 9:**
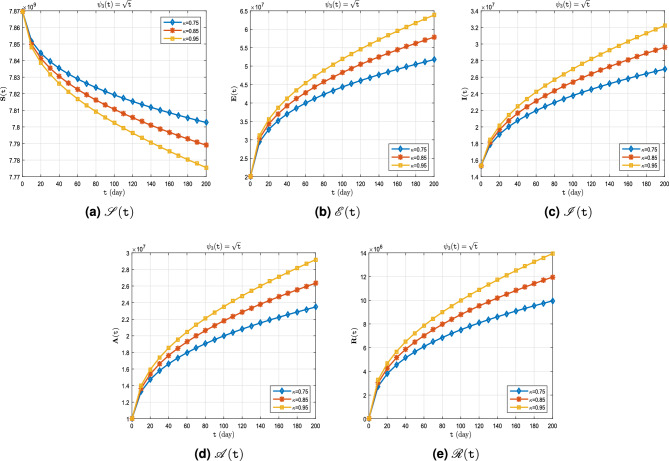
Dynamics of $\mathscr {S}(\texttt{t})$, $\mathscr {E}(\texttt{t})$, $\mathscr {I}(\texttt{t})$, $\mathscr {A}(\texttt{t})$ and $\mathscr {R}(\texttt{t})$ whenever $\uppsi _3(\texttt{t})= \sqrt{\texttt{t}}$ and different fractional order $\upkappa = 0.75, 0.85, 0.95$.

Similar to **Case I** and **Case II**, the real data for infected cases, as well as the results of model (8) for $\upkappa \in \{0.95, 1\}$ on period $\breve{T}$ can be seen in Fig. 8. Also, in Fig. 9, it is predicted how each of the classes $\mathscr {S}$, $\mathscr {E}$, $\mathscr {I}$, $\mathscr {A}$ and $\mathscr {R}$ will change with $\upkappa \in \{ 0.75, 0.85, 0.95\}$. In addition, Tables 9, 10 and 11 show these results. The graphs (Fig. 9a–e), in this case are remarkable. Over the course of time, only $\mathscr {S}$ decreases as the order of the derivative $\kappa$ approaches one (Fig. 9a), But the value of rest of the parameters $\mathscr {E}$, $\mathscr {I}$, $\mathscr {A}$ and $\mathscr {R}$ increases more whenever the order of the derivative $\kappa$ approaches one more (Fig. 9b–e).

**Table 9:** Obtained results of $\mathscr {S}(\texttt{t})$, $\mathscr {E}(\texttt{t})$, $\mathscr {I}(\texttt{t})$, $\mathscr {A}(\texttt{t})$ and $\mathscr {R}(\texttt{t})$ whenever $\uppsi _3(\texttt{t})= \sqrt{\texttt{t}}$ and fractional order $\upkappa = 0.75$.

$\texttt{t}$	$\uppsi _3(\texttt{t})= \sqrt{\texttt{t}}, \quad {\upkappa =0.75}$				
	$\mathscr {S}$	$\mathscr {E}$	$\mathscr {I}$	$\mathscr {A}$	$\mathscr {R}$
0	7869665900	20000000	15315220	10000000	0
1	7865452967	22496609	15748219	10636302	645391
2	7862675410	24000485	16111461	11122253	1068066
3	7860537499	25108429	16419469	11518472	1391902
4	7858771102	26000499	16687842	11855852	1658548
$\vdots$	$\vdots$	$\vdots$	$\vdots$	$\vdots$	$\vdots$
100	7819280579	44311816	23783634	19984670	7508561
101	7819073686	44404890	23822952	20028022	7538958
102	7818868032	44497393	23862045	20071116	7569172
103	7818663598	44589336	23900915	20113957	7599207
$\vdots$	$\vdots$	$\vdots$	$\vdots$	$\vdots$	$\vdots$
197	7803079926	51567958	26887359	23384206	9887173
198	7802940696	51630081	26914214	23413455	9907608
199	7802801862	51692024	26940995	23442621	9927986
200	7802663419	51753789	26967704	23471705	9948306

**Table 10:** Obtained results of $\mathscr {S}(\texttt{t})$, $\mathscr {E}(\texttt{t})$, $\mathscr {I}(\texttt{t})$, $\mathscr {A}(\texttt{t})$ and $\mathscr {R}(\texttt{t})$ whenever $\uppsi _3(\texttt{t})= \sqrt{\texttt{t}}$ and fractional order $\upkappa = 0.85$.

$\texttt{t}$	$\uppsi _3(\texttt{t})= \sqrt{\texttt{t}}, \quad {\upkappa =0.85}$				
	$\mathscr {S}$	$\mathscr {E}$	$\mathscr {I}$	$\mathscr {A}$	$\mathscr {R}$
0	7869665900	20000000	15315220	10000000	0
1	7865101369	22704969	15784356	10689406	699253
2	7862056800	24342361	16188564	11227239	1162342
3	7859693049	25556695	16535145	11670122	1520093
4	7857726442	26540676	16839313	12049767	1816643
$\vdots$	$\vdots$	$\vdots$	$\vdots$	$\vdots$	$\vdots$
100	7810600458	48222416	25428095	21801971	8785251
101	7810337886	48339940	25478453	21857098	8823797
102	7810076727	48456816	25528552	21911931	8862134
103	7809816957	48573056	25578396	21966474	8900267
$\vdots$	$\vdots$	$\vdots$	$\vdots$	$\vdots$	$\vdots$
197	7789550567	57604673	29495040	26226278	11875142
198	7789365380	57686914	29531036	26265235	11902335
199	7789180647	57768950	29566948	26304096	11929461
200	7788996363	57850782	29602775	26342864	11956523

**Table 11:** Obtained results of $\mathscr {S}(\texttt{t})$, $\mathscr {E}(\texttt{t})$, $\mathscr {I}(\texttt{t})$, $\mathscr {A}(\texttt{t})$ and $\mathscr {R}(\texttt{t})$ whenever $\uppsi _3(\texttt{t})= \sqrt{\texttt{t}}$ and fractional order $\upkappa = 0.95$.

$\texttt{t}$	$\uppsi _3(\texttt{t})= \sqrt{\texttt{t}}, \quad {\upkappa =0.95}$				
	$\mathscr {S}$	$\mathscr {E}$	$\mathscr {I}$	$\mathscr {A}$	$\mathscr {R}$
0	7869665900	20000000	15315220	10000000	0
1	7864823004	22869930	15812966	10731449	741896
2	7861559101	24616328	16251192	11312214	1238171
3	7859006095	25919245	16630434	11794449	1624321
4	7856869348	26980902	16965295	12210178	1946260
$\vdots$	$\vdots$	$\vdots$	$\vdots$	$\vdots$	$\vdots$
100	7802431105	51885500	26988854	23515767	9986045
101	7802111117	52028204	27050615	23583030	10033008
102	7801792671	52170203	27112091	23649970	10079744
103	7801475744	52311508	27173287	23716593	10126258
$\vdots$	$\vdots$	$\vdots$	$\vdots$	$\vdots$	$\vdots$
197	7776216847	63528039	32083114	29031122	13835586
198	7775981251	63632299	32129151	29080727	13870219
199	7775746148	63736335	32175095	29130229	13904782
200	7775511532	63840150	32220948	29179629	13939273

## Conclusion

In this paper, an epidemic model $\mathscr {S}\mathscr {E}\mathscr {I}\mathscr {A}\mathscr {R}\mathscr {S}$ for the transmission of infection caused via $\mathscr {C}$-19 is presented utilizing the $\uppsi$-Caputo derivative. The reason for utilizing the derivative of the fractional order is that it provides a more accurate fit than the derivative of the integer order. The reproduction number has been calculated and its sensitivity has also been explored. The EPs have been calculated, and their stability are investigated. We show that the model is locally and globally asymptotically stable if $\breve{\mathscr {R}}_0$ is less than 1. The existence and uniqueness of the solution for the model via the FP theorem has been proven. Utilizing the FEP, an approximate solution to the model has been calculated.

In addition, the number of infection cases in the world in the period of time $\breve{T}$, 15 June to 4 August 2022, is considered to simulate the model to the real information. Also, the behavior of each of the classes after August 4 to the next 150 days is predicted with different cases for $\uppsi$. System (8) have been simulated in three cases for $\uppsi (\texttt{t})$ as $\uppsi _1(\texttt{t}) = \texttt{t}$ (Caputo derivative); $\uppsi _2(\texttt{t}) = \ln \texttt{t}$ (Caputo–Hadamard derivative); $\uppsi _3(\texttt{t}) = \sqrt{\texttt{t}}$ (Katugampola derivative). In the simulation by $\uppsi _1(\texttt{t})$, as can be seen in Fig. 4, the designed model has very good support from the data. In the simulation by $\uppsi _2(\texttt{t})$, as can be seen in Fig. 6, the designed model has acceptable support from the data, and in the simulation by $\uppsi _3(\texttt{t})$, as can be seen in Fig. 8, the designed model has good support from the data. In all simulations, the advantage of utilizing the derivative of the fractional order instead of the utilizing of the integer order can be seen. In Figs. 5c, 7c and 9c, respectively, utilizing $\uppsi _1(\texttt{t})$, $\uppsi _2(\texttt{t})$ and $\uppsi _3(\texttt{t})$, as well as utilizing different values for $\upkappa$, the spread of the disease after August 4 is predicted. At the end, by comparing the simulation results and real data, we come to the conclusion that the simulation utilizing $\uppsi _1(\texttt{t})= \texttt{t}$ (Caputo derivative) with the order of 0.95 shows the prevalence of the disease better.

## Data Availability

The datasets used and/or analyzed during the current study available from the corresponding author on reasonable request.
